# Talazoparib plus enzalutamide versus olaparib plus abiraterone acetate and niraparib plus abiraterone acetate for metastatic castration-resistant prostate cancer: a matching-adjusted indirect comparison

**DOI:** 10.1038/s41391-024-00924-x

**Published:** 2024-12-07

**Authors:** Elena Castro, Di Wang, Sarah Walsh, Samantha Craigie, Anja Haltner, Jonathan Nazari, Alexander Niyazov, Imtiaz A. Samjoo

**Affiliations:** 1 Hospital Universitario 12 de Octubre, Madrid, Spain; 2 EVERSANA™, Burlington, ON Canada; 3 EVERSANA™, Chicago, IL USA; 4 Pfizer, Inc., New York, NY USA

**Keywords:** Prostate cancer, Prostate cancer

## Abstract

**Background:**

Without head-to-head trials between talazoparib+enzalutamide (TALA + ENZA), olaparib+abiraterone acetate (OLAP + AAP), and niraparib plus AAP (NIRA + AAP) the ability to evaluate their relative efficacy as first-line (1 L) treatment in metastatic castration-resistant prostate cancer (mCRPC) is limited. The objective of this study was to assess the relative efficacy between TALA + ENZA (TALAPRO-2) versus OLAP + AAP (PROpel) and NIRA + AAP (MAGNITUDE) in 1 L mCRPC via a matching-adjusted indirect treatment comparison (MAIC).

**Methods:**

Patient-level data from TALAPRO-2 and published data from PROpel and MAGNITUDE were used. TALAPRO-2 data were reweighted to satisfy the eligibility criteria for PROpel and MAGNITUDE. Talazoparib (0.5 mg/day) plus enzalutamide (160 mg/day) was compared to olaparib (300 mg twice daily) plus abiraterone acetate (1000 mg/day) and niraparib (200 mg/day) plus abiraterone acetate (1000 mg/day). Hazard ratios (HRs) were calculated for radiographic progression-free survival (rPFS) and overall survival (OS), and odds ratios (ORs) for prostate-specific antigen (PSA) response and objective response rate (ORR). Additional efficacy outcomes were assessed.

**Results:**

In all-comers, TALA + ENZA was statistically superior to OLAP + AAP for rPFS (HR: 0.727; 95% confidence interval [CI]: 0.565, 0.935) and PSA response (OR: 1.663; 1.101, 2.510), and numerically favored for OS (HR: 0.847; 0.667, 1.076) and ORR (OR: 1.109; 0.646, 1.903). In patients with homologous recombination repair mutations (HRRm), relative to NIRA + AAP, TALA + ENZA was statistically superior for rPFS (HR: 0.460; 0.280, 0.754), and numerically favored for OS (HR: 0.601; 0.347, 1.041) and ORR (OR: 1.524; 0.579, 4.016).

**Conclusions:**

Results suggest that TALA + ENZA may provide improvements in clinical outcomes relative to OLAP + AAP and NIRA + AAP in 1 L mCRPC; however, inherent limitations associated with the complexity of the analyses must be considered.

## Introduction

Prostate cancer is the second most common malignancy diagnosed among men globally [1]. Despite treatment advances, metastatic castration-resistant prostate cancer (mCRPC) remains an incurable, and deadly disease [2]. There is a need for mCRPC treatment options to extend survival, prevent disease progression, and improve or maintain quality of life.

Poly (ADP-ribose) polymerase inhibitors (PARPi) offer a promising new approach for long-term disease control in certain cancers. Several PARPi are currently being tested in clinical trials for the treatment of mCRPC patients. The randomized, double-blind, placebo-controlled, Phase 3 clinical trial (TALAPRO-2; NCT03395197) compared talazoparib plus enzalutamide (TALA + ENZA) to placebo plus enzalutamide in mCRPC patients. Two separate cohorts were enrolled based on HRR gene mutation status: Cohort 1 included all-comers whereas Cohort 2 included only patients with HRR gene mutations (HRRm) [3]. Treatment with TALA + ENZA demonstrated favorable results versus placebo plus enzalutamide in the all-comers population and in patients harboring HRR gene alterations [4, 5].

The randomized, double-blind, placebo-controlled, Phase 3 PROpel trial (NCT03732820) investigated olaparib plus abiraterone acetate (OLAP + AAP) versus placebo plus abiraterone acetate (AAP) as first-line mCRPC treatment in an all-comers population [6, 7]. Results of the planned primary analyses from the first data cutoff of the trial showed a significantly longer median rPFS by investigator assessment for OLAP + AAP versus placebo plus AAP [8, 9]. The randomized, double-blind, placebo-controlled, Phase 3 MAGNITUDE trial (NCT03748641) investigated niraparib plus abiraterone acetate (NIRA + AAP) versus placebo plus abiraterone acetate (AAP) as first-line treatment in patients with mCRPC and biomarker positive for HRR gene mutations (HRR BM + ). In the HRR BM+ cohort, rPFS assessed by central review was significantly longer in the NIRA + AAP group compared with the placebo plus AAP group [10].

**Fig. 1:**
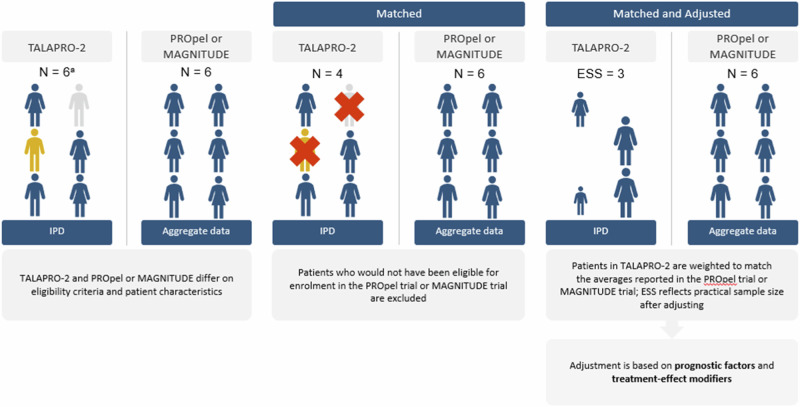
Overview of matching and adjusting steps in a matching-adjusted treatment comparison. ^a^Note that the n’s presented in this figure are for illustrative purposes only. ESS effective sample size; IPD individual patient data. Source: this figure was adapted from Rugo et al. [31] with permission.

Comparisons between these therapies are of particular interest, given that these PARPi share a similar mechanism of action, but their comparative efficacy and safety has not been assessed in head-to-head trials. Indirect treatment comparisons (ITCs) allow for the comparison of two or more interventions using data from each of their respective studies to estimate the relative effects of intervention in the absence of head-to head trials. When performing ITCs, it is important to assess the similarity of the studies involved in the analysis. Standard ITC methods such as network meta-analysis or Bucher analysis use aggregate data from published trials that share common comparators, assuming similarity and homogeneity between randomized controlled trials (RCTs), to compare relative effects between treatments. When an imbalance in treatment effect modifiers is evident and individual patient-level data (IPD) are available from at least one trial (i.e., TALAPRO-2), population-adjusted indirect comparison methods like matching-adjusted indirect comparisons (MAICs) can be used to adjust for these imbalances and estimate a less biased relative treatment effect than standard indirect treatment comparisons. Using IPD from one study (i.e., TALAPRO-2), patients are selected to match the eligibility criteria of the comparator study (i.e., PROpel or MAGNITUDE), and any remaining differences are adjusted by reweighting patients in the study with available IPD (i.e., TALAPRO-2) to make the populations more similar with respect to key baseline characteristics (see Fig. 1).

The objective of this analysis was to estimate the relative efficacy of patients randomized to TALA + ENZA in TALAPRO-2 versus OLAP + AAP in PROpel (all-comers) and NIRA + AAP in MAGNITUDE (HRRm) for the treatment of 1 L patients with mCRPC through MAICs.

## Materials and methods

### Systematic literature search

Relevant RCTs evaluating 1 L treatments in men ≥18 years with asymptomatic or mildly symptomatic mCRPC were identified via a systematic literature review (SLR) following established best practices [6, 11]. The protocol was registered with the International Prospective Register of Systematic Reviews (registration number: CRD42021283512).

The search strategy was developed and tested by a medical information specialist in consultation with the review team and peer-reviewed independently by another senior medical information specialist using the Peer Review of Electronic Search Strategies (PRESS) checklist. The following electronic databases were searched using the Ovid® search interface: Embase, Ovid MEDLINE® (including Epub Ahead of Print and In-Process & Other Non-Indexed Citations), Ovid MEDLINE® Daily, Cochrane Central Register of Controlled Trials, and the Cochrane Database of Systematic Reviews. These searches were performed on September 9, 2021, and updates of these searches were performed on February 17, 2022, and October 3, 2022.

Websites of key clinical conferences were hand searched for relevant abstracts from 2019 to 2022. Grey literature searches of key clinical conferences, the US National Institutes of Health (NIH) ClinicalTrials.gov, Food and Drug Administration (FDA), European Medicines Agency (EMA), and reference lists of previously published reviews were conducted. Key HTA agencies (National Institute for Health and Care Excellence [NICE], Canadian Agency for Drugs and Technologies in Health [CADTH], and Scottish Medicines Agency [SMC]) were also hand searched for relevant technology appraisals.

Title and abstract screening against pre-defined eligibility criteria was performed in duplicate by two reviewers using DistillerSR [7]. After initial screening, full-text articles were obtained for potentially eligible citations and reviewed by two reviewers to determine formal inclusion. Reasons for exclusion were documented at the full-text stage. Any screening discrepancies between the two reviewers were resolved by consensus or by a third independent reviewer. Detailed study eligibility criteria is available in Supplementary Appendix A. Data extraction was performed by one reviewer and validated by a second reviewer using data extraction templates in Microsoft® Excel (Microsoft Corporation, Seattle, US). Data elements captured during extraction included publication characteristics, study setting, study methods, study participants, and study findings. The risk of bias assessment was performed using the NICE Single Technology Appraisal Evidence Submission Checklist for assessment of risk of bias in RCTs [12].

### Feasibility assessment

To ensure sensible and robust comparisons in the indirect treatment analysis, the similarity of included studies of interest was qualitatively assessed. Between-study heterogeneity was conducted for the following elements: trial design and methodology, baseline patient characteristics, trial eligibility criteria, outcome assessments, interventions, comparators, and their contribution to a connected network (i.e., a common comparator).

### Outcomes

Outcomes assessed in this analysis included rPFS as assessed by blinded independent central review (BICR), overall survival (OS), PFS on the next line of therapy (PFS2), time to prostate-specific antigen (PSA) progression, time to cytotoxic chemotherapy initiation, PSA response, and objective response rate (ORR).

### Statistical analysis

#### Matching-adjusted Indirect comparison

Given the lack of a common comparator between TALAPRO-2 and PROpel, and TALAPRO-2 and MAGNITUDE, unanchored MAICs were conducted to estimate the relative treatment effect of TALA + ENZA versus OLAP + AAP, and NIRA + AAP by leveraging IPD from TALAPRO-2 (all-comers for comparisons with PROpel and HRRm for comparisons with MAGNITUDE) and published aggregate data from PROpel (all-comers) and MAGNITUDE (HRRm).

The phase 3 TALAPRO-2 trial was assessing TALA + ENZA as 1 L treatment in patients with mCRPC in two cohorts: Cohort 1, the all-comers cohort and Cohort 2, HRR-deficient only for DNA damage response alterations in genes directly or indirectly involved in HRR [4]. The most recent data cutoff (DCO) for TALAPRO-2 Cohort 1 (DCO: 28/03/23 for OS and DCO: 16/08/22 for all other outcomes) and TALAPRO-2 Cohort 2 (DCO: 03/10/22 for all outcomes) were used for the present analysis. Analysis of comparator studies also used the most mature data identified in the searches. For the PROpel trial, this analysis used results based on DCO 12/10/22 for OS and PFS2 [13], DCO 14/03/22 for ORR [14], and DCO 30/07/21 for rPFS (BICR) and PSA response [8]. For the MAGNITUDE trial, this analysis used results based on DCO 17/06/2022 for rPFS (BICR), OS and time to cytotoxic chemotherapy [10] and DCO 8/10/2021 for both time to PSA progression and ORR [15]. Note, data for TALA + ENZA was provided by Pfizer at the time of review given the TALAPRO-2 trial was ongoing. Since the last SLR update conducted in October 2022, results for the TALAPRO-2 trial have been published (DCO: 16/08/22) [4]. Updated OS data (DCO: 28/03/23) for TALAPRO-2 used in the present analysis were provided by Pfizer (confidential, data on file). Updated data for PROpel [13] and MAGNITUDE [16], and additional information from the full-text publications for MAGNITUDE were incorporated into the analyses. Data were derived from aggregate data for PROpel and MAGNITUDE by reconstructing reported data for PSA response and ORR, and by simulating IPD for rPFS, OS, PFS2, time to PSA progression, and time to cytotoxic chemotherapy initiation from digitized published Kaplan-Meier (KM) curves using the Guyot method [17–19].

The TALAPRO-2 population was adjusted to match the eligibility criteria and distribution of prognostic and treatment effect-modifying factors between study populations. Patients from TALAPRO-2 who did not fulfill the eligibility criteria of the comparator study were removed to better align the two populations. Patients from TALAPRO-2 who satisfied the eligibility criteria of PROpel and MAGNITUDE, respectively, were reweighted to adjust for imbalances in baseline characteristics of prognostic significance (see Appendix B). Factors predictive of the treatment effect of therapies in mCRPC were identified through the literature [20] and clinical expertise and were ranked in order of importance by an experienced clinician (see Supplementary Table 2). Population differences between TALAPRO-2 and the comparator studies were assessed using standardized mean differences (SMDs), where an SMD between 0 and 0.1 was considered a small difference, an SMD > 0.1 and ≤0.2 was a moderate difference, and an SMD of >0.2 was a substantial difference [21].

A form of propensity score weighting was used, in which patients in TALAPRO-2 were weighted by the inverse odds of being in that group compared to the other group based on the values of their baseline characteristics. Weights were based on a generalized method-of-moments propensity score algorithm, which guaranteed a close balancing of covariates between TALAPRO-2 and PROpel populations, and TALAPRO-2 and MAGNITUDE populations [22, 23]. Results of the TALAPRO-2 study were reanalyzed using the weighted patient-level dataset. Treatment outcomes were then compared across balanced study populations.

The effective sample size (ESS) was calculated to reflect the impact of weighting on the available information in the IPD [22]. The ESS is the number of non-weighted patients that would produce a treatment effect estimate with the same precision as the reweighted sample estimate. Since these MAIC analyses provided an unanchored indirect comparison due to the lack of a common comparator arm in each comparison, all treatment effect modifiers and prognostic variables should be adjusted to ensure balance and reduce bias [22].

For time-to-event outcomes (i.e., rPFS, OS, PFS2, time to PSA progression, time to cytotoxic chemotherapy initiation), hazard ratios (HRs) were estimated using a weighted Cox proportional hazards model. For response outcomes (i.e., PSA response, ORR) odds ratios (ORs) were estimated using a weighted generalized linear regression model using a binomial likelihood and logit link function. The corresponding variances were estimated using a robust sandwich estimator, and 95% confidence intervals (CIs) were reported.

Validity of the proportional hazards assumption for rPFS, OS, PFS2, time to PSA progression, and time to cytotoxic chemotherapy initiation was assessed using the Grambsch-Therneau test [24] (with a p-value less than. 05 considered to indicate a violation of the assumption).

Additional unanchored MAICs for rPFS and OS were conducted to estimate the relative treatment effects between the control arm from TALAPRO-2 (ENZA, all-comers) using IPD and both the active and control arms from PROpel (OLAP + AAP and abiraterone acetate plus placebo [AAP + PBO]) using published aggregate data as well as TALA + ENZA (TALAPRO-2) versus AAP + PBO (PROpel). Similarly, unanchored MAICs for rPFS and OS were conducted to estimate the relative treatment effects between the control arm from TALAPRO-2 Cohort 2 (ENZA) using IPD and both the active and control arms from MAGNITUDE (NIRA + AAP and AAP + PBO) as well as TALA + ENZA (TALAPRO-2 Cohort 2) versus AAP + PBO (MAGNITUDE). The same statistical methodology as described above was applied for these analyses. All analyses were conducted using R version 4.1.2 (R Core Team, Vienna, Austria) based on the methods developed by Signorovitch et al. [25], and as implemented by the National Institute for Health and Care Excellence (NICE) Decision Support Unit Technical Support Document series [26].

#### Sensitivity analysis

Sensitivity analyses were conducted to investigate the impact on the treatment effect estimates, ESS, and SMD when adjusting for additional covariates in the analysis. A series of sensitivity analyses were conducted whereby all commonly available rank-ordered variables were sequentially and incrementally adjusted for until the final model contained all available factors.

#### Exploratory analysis

In addition to the twelve prognostic factors identified which informed the primary analysis, four additional covariates were identified as variables of interest (i.e., *BRCA1*, *BRCA2*, *BRCA* co-occurring, and *PALB2*), based on clinical opinion. A series of exploratory analyses were also conducted whereby these additional factors were sequentially and incrementally adjusted for.

## Results

### Systematic literature review and feasibility assessment

Three RCTs were identified and were the primary focus for this analysis (Table 1). Included studies were of good quality (Supplementary Table 4). Although there were some differences in trial design and methodology, patient populations, trial enrollment criteria, and outcome assessments, the three trials were deemed sufficiently similar, and supported by clinical opinion, to derive reasonable estimates of comparative efficacy via MAICs (Supplementary Appendix E**)**.

**Table 1:** Summary of included randomized controlled trials.

Trial; NCT	Phase	Setting	Blinding	Treatment arms	N enrolled
TALAPRO-2; NCT03395197	3	Multicenter	Double-blind	Talazoparib 0.5 mg Enzalutamide 160 mg	Cohort 1 (all-comers): 402 Cohort 2 (HRR BM + ): 200
				Placebo Enzalutamide 160 mg	Cohort 1 (all-comers): 403 Cohort 2 (HRR BM + ): 199
PROpel; NCT03732820^a^	3	Multicenter	Double-blind	Olaparib 300 mg twice daily Abiraterone 1000 mg Prednisone/ Prednisolone 5 mg twice daily	399
				Placebo Abiraterone 1000 mg Prednisone/ Prednisolone 5 mg twice daily	397
MAGNITUDE; NCT03748641^b^	3	Multicenter	Double-blind	Niraparib 200 mg Abiraterone acetate 1000 mg Prednisone 10 mg	212 HRR BM+
				Placebo Abiraterone acetate 1000 mg Prednisone 10 mg	211 HRR BM+

^a^Patients in the PROpel trial consisted of 25.8% in the olaparib + abiraterone acetate arm and 20.2% in the placebo + abiraterone acetate arm who were symptomatic (defined as those with a BPI-SF score ≥4 and/or opiate use).

^b^Data presented are from the HRR BM+ cohort since limited data were reported for the HRR BM- cohort. Patients in the MAGNITUDE trial consisted of 23.6% in the niraparib + abiraterone acetate arm and 22.7% in the placebo + abiraterone acetate arm who received ≤4 months of abiraterone acetate therapy for first-line mCRPC.

*AAP* abiraterone acetate plus prednisone/prednisolone, *BM* biomarker, *BPI-SF* Brief Pain Inventory - Short Form, *HRR* homologous recombination repair, *mCRPC* metastatic castration-resistant prostate cancer, *mg* milligram, *N* number of patients.

### TALAPRO-2 versus PROpel (All-comers)

#### Adjustment of prognostic factors and population alignment

Prior to adjustment, the observed baseline characteristics (except for Eastern Cooperative Oncology Group Performance Status [ECOG PS], Gleason score, and alkaline phosphatase [ALP] levels) were similar between the 402 patients assigned to receive TALA + ENZA and the 399 patients assigned to receive OLAP + AAP. Of the two populations, TALAPRO-2 had a greater proportion of patients with ECOG PS = 1 and Gleason score ≥ 8, and higher mean ALP levels, whereas PROpel had a greater proportion of patients with bone metastases. In the adjusted analysis, removal of patients in TALAPRO-2 was not required, as key trial eligibility criteria were similar between the trials or broader in the PROpel trial (i.e., Brief Pain Index – Short Form [BPI-SF]). After reweighting, all baseline characteristics of interest aligned exactly across the two populations, accompanied by a 25% reduction in ESS (Table 2). Results for rPFS, OS and ORR are reported herein. Results for PFS2, and PSA response are available in Appendix F.

**Table 2:** Unadjusted and adjusted baseline characteristics in TALAPRO-2 (TALA + ENZA) and PROpel (OLAP + AAP).

Characteristic	PROpel	TALAPRO-2 Cohort 1 (All-comers)														
		Naïve	Stratification factors			Number of factors										
			1	2	3	1	2	3	4	5	6	7	8	9	10	11
														Primary	Exploratory	
HRR alteration status (%)	27.8%	21.1%	27.8%	27.8%	27.8%	27.8%	27.8%	27.8%	27.8%	27.8%	27.8%	27.8%	27.8%	27.8%	27.8%	27.8%
Prior NHT (%)	0.3%	5.7%	6.1%	0.3%	0.3%	0.3%	0.3%	0.3%	0.3%	0.3%	0.3%	0.3%	0.3%	0.3%	0.3%	0.3%
Prior taxane (%)	24.3%	21.4%	21.3%	22.4%	24.3%	24.3%	24.3%	24.3%	24.3%	24.3%	24.3%	24.3%	24.3%	24.3%	24.3%	24.3%
Liver metastases (%)	3.8%	3.0%	2.8%	2.4%	2.4%	3.8%	3.8%	3.8%	3.8%	3.8%	3.8%	3.8%	3.8%	3.8%	3.8%	3.8%
Bone metastases (%)	87.5%	84.3%	84.4%	83.4%	83.3%	83.4%	87.5%	87.5%	87.5%	87.5%	87.5%	87.5%	87.5%	87.5%	87.5%	87.5%
ECOG = 1 (%)	28.1%	35.6%	35.4%	34.6%	34.5%	34.7%	34.8%	28.1%	28.1%	28.1%	28.1%	28.1%	28.1%	28.1%	28.1%	28.1%
PSA > 17.9 μg/L (%)^a^	50.0%	50.5%	50.9%	50.4%	50.4%	50.7%	51.2%	50.4%	50.0%	50.0%	50.0%	50.0%	50.0%	50.0%	50.0%	50.0%
Gleason score ≥ 8 (%)	66.4%	69.9%	70.9%	70.5%	70.7%	70.9%	71.2%	71.8%	71.7%	66.4%	66.4%	66.4%	66.4%	66.4%	66.4%	66.4%
HGB (mean [SD]) ^b^	130.5 (12.8)	129.6 (13.8)	129.5 (13.8)	129.5 (13.8)	129.5 (13.8)	129.3 (13.9)	129.3 (13.9)	129.5 (13.8)	129.4 (13.7)	129.6 (13.7)	130.5 (12.8)	130.5 (12.8)	130.5 (12.8)	130.5 (12.8)	130.5 (12.8)	130.5 (12.8)
LDH^b,c^ (mean [SD])	3.9 (1.3)	4.2 (3)	4.2 (2.9)	4.1 (2.2)	4.1 (2.2)	4.1 (2.2)	4.2 (2.3)	4.1 (2.2)	4.1 (2.2)	4.1 (2.2)	4.1 (2.1)	3.9 (1.3)	3.9 (1.3)	3.9 (1.3)	3.9 (1.3)	3.9 (1.3)
Albumin (mean [SD])^b^	42.4 (3.9)	41.8 (3.9)	41.8 (3.9)	41.8 (3.9)	41.8 (3.9)	41.8 (4)	41.8 (4)	41.8 (4)	41.8 (4)	41.8 (3.9)	41.9 (3.9)	42 (3.9)	42.4 (3.9)	42.4 (3.9)	42.4 (3.9)	42.4 (3.9)
ALP (mean [SD])^b^	2.6 (2.1)	3.6 (5.8)	3.7 (6.1)	3.7 (6.3)	3.6 (6.2)	3.7 (6.2)	3.8 (6.4)	3.8 (6.5)	3.8 (6.5)	3.8 (6.4)	3.7 (6.2)	3.6 (6.3)	3.6 (6.2)	2.6 (2.1)	2.6 (2.1)	2.6 (2.1)
BRCA1 (%)	2.3%	0.7%	1.0%	1.1%	1.1%	1.1%	1.0%	1.1%	1.2%	1.1%	1.2%	1.3%	1.4%	1.5%	2.3%	2.3%
BRCA2 (%)	9.5%	5.0%	6.5%	6.9%	6.9%	6.9%	7.0%	7.0%	7.1%	7.0%	6.8%	6.9%	6.8%	6.8%	6.5%	9.5%
*N or ESS*	*N* = *399*	*ESS* = *402*	*ESS* = *392*	*ESS* = *368*	*ESS* = *367*	*ESS* = *364*	*ESS* = *360*	*ESS* = *353*	*ESS* = *351*	*ESS* = *342*	*ESS* = *339*	*ESS* = *323*	*ESS* = *316*	*ESS* = *300*	*ESS* = *298*	*ESS* = *290*
*Mean SMD*		*0.131*	*0.117*	*0.088*	*0.084*	*0.082*	*0.078*	*0.065*	*0.062*	*0.054*	*0.044*	*0.035*	*0.028*	*0.011*	*0.008*	*0.000*

^a^PSA was converted to a dichotomous variable and used the PROpel median PSA as the cut-off (17.9 μg/L) since the MAIC failed to converge with the continuous variable. To achieve half of the population with PSA levels below the median and half with PSA levels above median, the sample size of PROpel was set to 398 patients for this variable.

^b^The mean and standard deviation were estimated from the reported median and interquartile range using the *estmeansd* package in R [30].

^c^PROpel reported in μkat/L whereas TALAPRO-2 used U/L. A conversion of 1 µkat/L = 60 U/L was performed to match the unit used in PROpel.

*ALP* alkaline phosphatase level, *ECOG* Eastern Cooperative Oncology Group, *ESS* effective sample size, *HGB* hemoglobin level, *HRR* homologous recombination repair, *LDH* lactate dehydrogenase level, *NHT* novel hormonal therapy, *PSA* prostate specific antigen, *SD* standard deviation, *SMD* standardized mean difference.

#### Radiographic progression-free survival

**Fig. 2:**
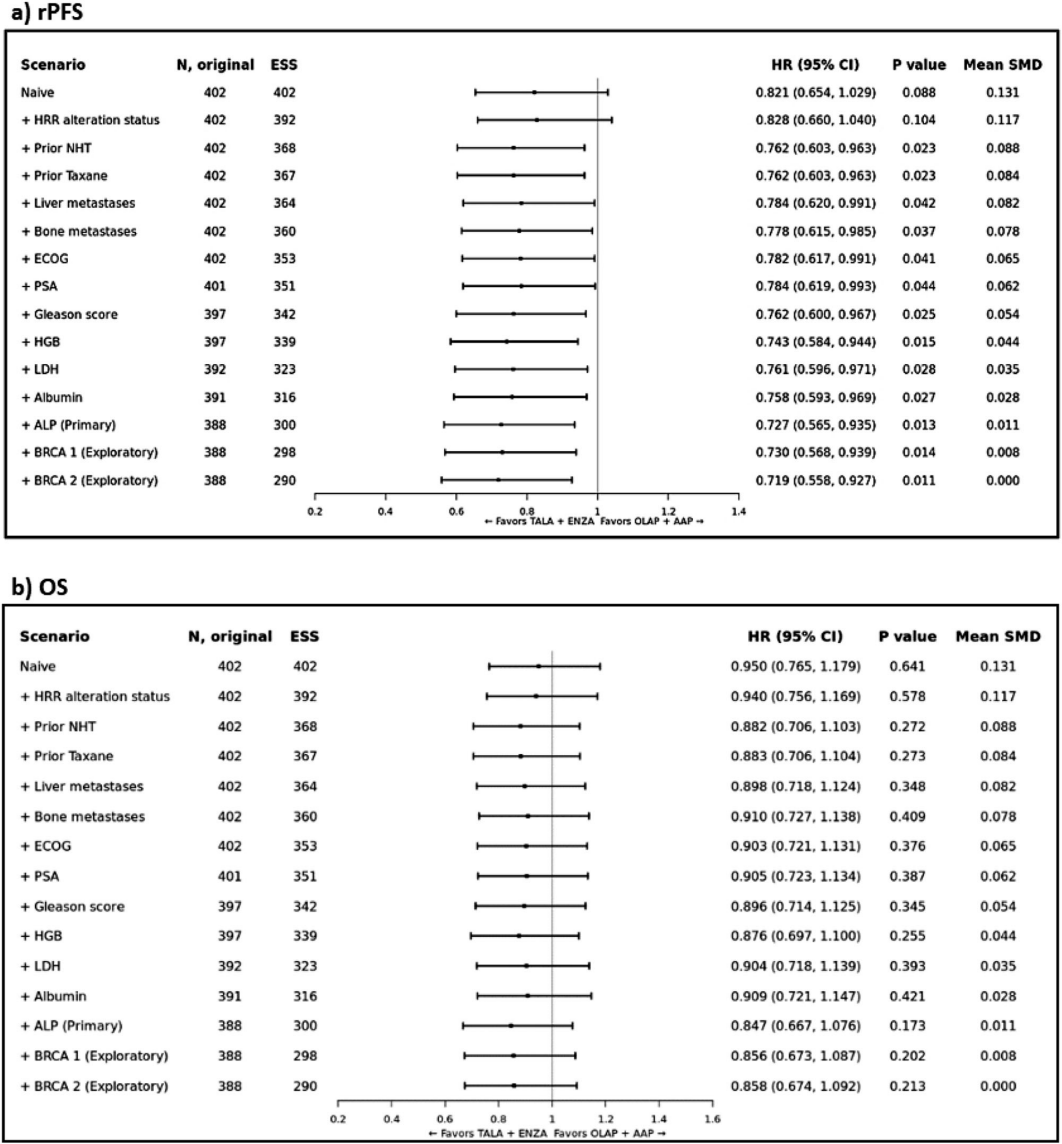
Summary of TALAPRO-2 (TALA + ENZA) and PROpel (OLAP + AAP) MAIC Results (All-comers). Forest plot of MAIC results for TALAPRO-2 (TALA + ENZA) and PROpel (OLAP + AAP) in the all-comers population for the outcome of **a**) rPFS; and **b**) OS. Note: rPFS was assessed by BICR. Note: Results of the naïve analysis and subsequent analyses which adjust for each new characteristic incrementally are shown. The primary analysis is indicated which adjusts for all factors listed above it and ALP. Patients with missing values for a given characteristic were excluded from the corresponding analysis. Note: An HR below 1.0 indicates an improved outcome for TALA + ENZA relative to OLAP + AAP. ALP alkaline phosphatase level, BICR blinded independent central review, CI confidence interval, ECOG Eastern Cooperative Oncology Group, ESS effective sample size, HGB hemoglobin level, HR hazard ratio, HRR homologous recombinant repair, LDH lactate dehydrogenase level, MAIC matching-adjusted indirect comparison, NHT novel hormonal therapy, OLAP + AAP olaparib plus abiraterone acetate; PSA prostate specific antigen, rPFS radiographic progression-free survival, SMD standardized mean difference; TALA + ENZA talazoparib plus enzalutamide.

Prior to adjustment (i.e., in the naïve analysis), the HR for TALA + ENZA versus OLAP + AAP was 0.821 (95% CI: 0.654, 1.029; *p* = 0.088) for rPFS. In the primary analysis, TALA + ENZA significantly improved rPFS (HR: 0.727; 95% CI: 0.565, 0.935; *p* = 0.013) compared to OLAP + AAP (Fig. 2a and Supplementary Fig. 7). Both sensitivity analyses that incrementally and sequentially adjusted for baseline covariates mutually reported in both trials and exploratory analyses showed consistent results with the primary analysis (Fig. 2a). Additional MAIC results (TALA + ENZA vs AAP + PBO, ENZA vs OLAP + AAP and ENZA vs AAP + PBO) are available in Appendix H.

#### Overall survival

Prior to adjustment, the HR for TALA + ENZA versus OLAP + AAP was 0.950 (95% CI: 0.765, 1.179; *p* = 0.641) for OS. In the primary analysis, the estimated treatment effect was in favor of TALA + ENZA (HR: 0.847; 95% CI: 0.667, 1.076; *p* = 0.173) compared to OLAP + AAP but not statistically significant (Fig. 2b and Supplementary Fig. 8). Both sensitivity analyses that incrementally and sequentially adjusted for baseline covariates mutually reported in both trials and exploratory analyses showed consistent results with the primary analysis (Fig. 2b). Additional MAIC results (TALA + ENZA vs AAP + PBO, ENZA vs OLAP + AAP and ENZA vs AAP + PBO) are available in Appendix H.

#### Objective response rate

Prior to adjustment, the OR for TALA + ENZA versus OLAP + AAP was 1.118 (95% CI: 0.687, 1.818; *p* = 0.653) for ORR. In the primary analysis, the estimated treatment effect was in favor of TALA + ENZA (OR: 1.109; 95% CI: 0.646, 1.903; *p* = 0.708) compared to patients treated with OLAP + AAP but not statistically significant (Supplementary Fig. 3). Both sensitivity analyses that incrementally and sequentially adjusted for baseline covariates mutually reported in both trials and exploratory analyses showed consistent results with the primary analysis (Supplementary Fig. 3).

### TALAPRO-2 versus MAGNITUDE (HRRm population)

#### Adjustment of prognostic factors and population alignment

TALAPRO-2 (Cohort 2, HRR-deficient only; *n* = 200) and the MAGNITUDE population (*n* = 212) included patients with DNA damage response alterations in genes directly or indirectly involved in HRR. Importantly, there were differences between the trials in the assessment for the alteration status of DNA damage response genes (Table 3). In the adjusted analysis, patients were excluded if the HRR gene was not assessed in the MAGNITUDE trial (Table 3). Additional removal of patients was not required as key trial eligibility criteria were similar between the trials or broader in the MAGNITUDE trial. After aligning the two populations on HRR status, and prior to adjustment, the observed baseline characteristics (except for liver metastases, PSA levels, Gleason score, lactate dehydrogenase [LDH] levels, ALP levels) were similar between the 176 patients assigned to receive TALA + ENZA and the 212 patients assigned to receive NIRA + AAP. Of the two populations, TALAPRO-2 had a higher proportion of patients with Gleason score ≥8 and higher LDH and ALP levels, whereas MAGNITUDE had a greater proportion of patients with liver metastases, and higher PSA levels. After reweighting, all baseline characteristics of interest aligned exactly across the two populations, accompanied by a 70% reduction in ESS for the TALA + ENZA arm (Table 4). Results for rPFS, OS and ORR are reported herein. Results for time to cytotoxic chemotherapy initiation and time to PSA progression are available in Appendix F.

**Table 3:** HRR gene alterations in TALAPRO-2 and MAGNITUDE.

HRR Gene Alteration	Available in TALAPRO-2	Available in MAGNITUDE	Included HRR Gene Alteration in Analysis
ATM	Yes	Yes	Yes
ATR	Yes	No	No
BRCA1	Yes	Yes	Yes
BRCA2	Yes	Yes	Yes
BRIP1	No	Yes	No
CDK12	Yes	Yes	Yes
CHEK2	Yes	Yes	Yes
FANCA	Yes	Yes	Yes
HDAC2	No	Yes	No
PALB2	Yes	Yes	Yes
MLH1	Yes	No	No
MRE11A	Yes	No	No
NBN	Yes	No	No
RAD51C	Yes	No	No

*HRR* homologous recombinant repair.

**Table 4:** Unadjusted and adjusted baseline characteristics in TALAPRO-2 (TALA + ENZA) and MAGNITUDE (NIRA + AAP).

Characteristic	MAGNITUDE (HRR BM + )	TALAPRO-2 Cohort 2 (HRR BM + )														
		Naïve	Stratification Factors		Number of Factors											
			1	2	1	2	3	4	5	6	7	8	9	10	11	12
												Primary	Exploratory			
Prior NHT (%)	3.80%	9.10%	3.80%	3.80%	3.80%	3.80%	3.80%	3.80%	3.80%	3.80%	3.80%	3.80%	3.80%	3.80%	3.80%	3.80%
Prior Taxane (%)	19.30%	27.30%	28.90%	19.3%	19.3%	19.3%	19.3%	19.3%	19.3%	19.3%	19.3%	19.3%	19.3%	19.3%	19.3%	19.3%
Liver metastases (%)	8.50%	4.50%	4.40%	4.80%	8.50%	8.50%	8.50%	8.50%	8.50%	8.50%	8.50%	8.50%	8.50%	8.50%	8.50%	8.50%
Bone metastases (%)	86.30%	85.20%	84.40%	84.40%	85.00%	86.3%	86.3%	86.3%	86.3%	86.3%	86.3%	86.3%	86.3%	86.3%	86.3%	86.3%
ECOG = 1 (%)	38.70%	36.90%	36.20%	35.60%	35.90%	36.00%	38.7%	38.7%	38.7%	38.7%	38.7%	38.7%	38.7%	38.7%	38.7%	38.7%
PSA^a^ (mean [SD])	160.1 (389.6)	76.8 (273.4)	75.9 (279.3)	78.9 (295.8)	100.7 (385.4)	101.2 (385.6)	99.9 (377.1)	160.1 (391)	160.1 (391)	160.1 (391.2)	160.1 (391.6)	160.1 (391.6)	160.1 (391.6)	160.1 (391.7)	160.1 (391.8)	160.1 (391.8)
Gleason score ≥ 8 (%)	68.20%	74.40%	74.40%	73.70%	74.80%	74.90%	74.50%	72.50%	68.2%	68.2%	68.2%	68.2%	68.2%	68.2%	68.2%	68.2%
HGB^a^ (mean [SD])	127.8 (18.8)	127.6 (13.7)	127.8 (13.7)	127.9 (13.8)	127.5 (14.1)	127.4 (14.1)	127.3 (14.1)	124.8 (14.9)	125.1 (14.9)	127.8 (18.8)	127.8 (18.9)	127.8 (18.9)	127.8 (18.9)	127.8 (18.9)	127.8 (18.9)	127.8 (18.9)
LDH^a^ (mean [SD])	210.7 (76.2)	243.4 (132.9)	244.3 (136.2)	243.5 (134.5)	250.5 (148.2)	251.2 (148.7)	251 (148.2)	265.2 (178.3)	261.4 (176.1)	258.4 (161)	210.7 (76.6)	210.7 (76.6)	210.7 (76.6)	210.7 (76.7)	210.7 (76.7)	210.7 (76.7)
ALP^a, b^ (mean [SD])	175.2 (196.2)	196.9 (392.5)	194 (399.9)	194.4 (422.7)	199.4 (418.1)	200.9 (421.3)	198.3 (413.3)	223 (418.8)	216.7 (401)	210.2 (347.2)	160.2 (155.8)	175.2 (197.3)	175.2 (197.2)	175.2 (197.3)	175.2 (197.3)	175.2 (197.4)
BRCA1 (%)	5.70%	4.50%	4.80%	4.60%	5.00%	4.90%	5.00%	5.10%	5.20%	5.60%	4.70%	4.50%	5.70%	5.70%	5.70%	5.70%
BRCA2 (%)	40.60%	31.20%	31.60%	31.10%	31.10%	31.30%	31.10%	29.80%	27.80%	27.70%	26.80%	27.6%	27.3%	40.6%	40.6%	40.6%
BRCA co-occurring (%)	7.50%	3.40%	3.20%	3.70%	3.50%	3.50%	3.70%	3.20%	3.70%	3.10%	4.40%	4.20%	4.10%	2.60%	7.50%	7.50%
PALB2 (%)	3.80%	3.40%	3.60%	3.60%	4.90%	4.90%	4.90%	4.40%	4.60%	4.00%	3.00%	2.90%	2.80%	2.30%	2.00%	3.80%
*N or ESS*	*N* = *212*	*ESS* = *176*	*ESS* = *171*	*ESS* = *163*	*ESS* = *158*	*ESS* = *158*	*ESS* = *158*	*ESS* = *106*	*ESS* = *97*	*ESS* = *79*	*ESS* = *55*	*ESS* = *53*	*ESS* = *53*	*ESS* = *50*	*ESS* = *48*	*ESS* = *48*
*Mean SMD*		*0.136*	*0.124*	*0.104*	*0.095*	*0.093*	*0.089*	*0.096*	*0.083*	*0.070*	*0.042*	*0.038*	*0.035*	*0.022*	*0.007*	*0.000*

^a^The mean and standard deviation were estimated from the reported median and range using the *estmeansd* package in R [30].

^b^MAGNITUDE reported in U/L whereas TALAPRO-2 used μkat/L. A conversion of 1 µkat/L = 60 U/L was performed to match the unit used in MAGNITUDE.

*CI* confidence interval, *ECOG* Eastern Cooperative Oncology Group, *ESS* effective sample size, *HGB* hemoglobin level, *HR* hazard ratio, *HRR BM* + = homologous recombinant repair biomarker positive, *LDH* lactate dehydrogenase level, *MAIC* matching-adjusted indirect comparison, *PSA* prostate specific antigen, *SD* standard deviation, *SMD* standardized mean difference.

#### Radiographic progression-free survival

**Fig. 3:**
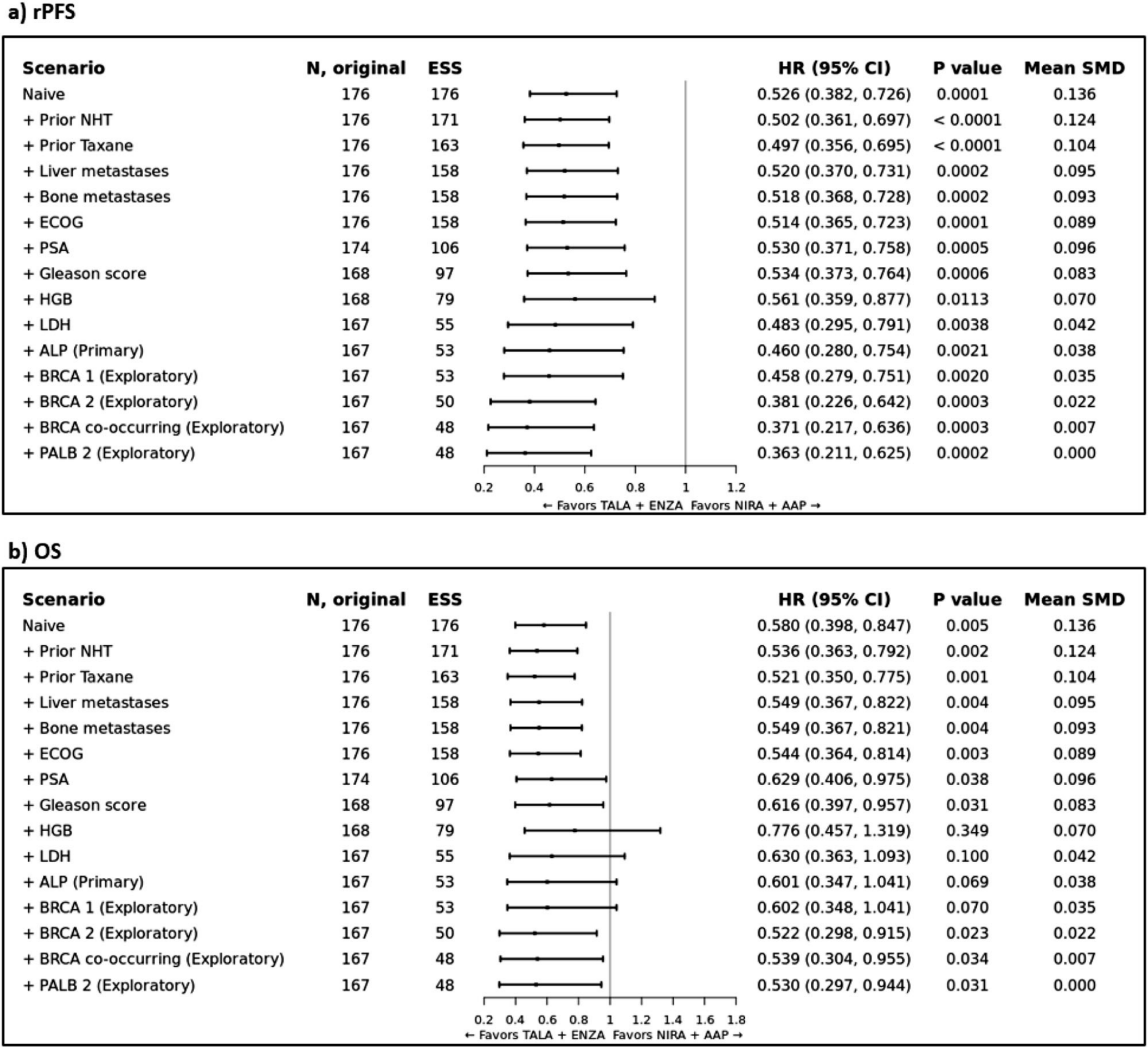
Summary of TALAPRO-2 (TALA + ENZA) and MAGNITUDE (NIRA + AAP) MAIC Results (HRR BM + ). Forest plot of MAIC results for TALAPRO-2 (TALA + ENZA) and MAGNITUDE (NIRA + AAP) in the HRR BM+ population for the outcome of **a**) rPFS; and **b**) OS. Note: rPFS was assessed by BICR. Note: Results of the naïve analysis and subsequent analyses which adjust for each new characteristic incrementally are shown. The primary analysis is indicated which adjusts for all factors listed above it and ALP. Patients with missing values for a given characteristic were excluded from the corresponding analysis. Note: An HR below 1.0 indicates an improved outcome for TALA + ENZA relative to NIRA + AAP. BICR blinded independent central review; CI confidence interval; ECOG = Eastern Cooperative Oncology Group; ESS = effective sample size; HGB = hemoglobin level; HR hazard ratio, HRR BM + = homologous recombination repair biomarker positive, LDH lactate dehydrogenase level, MAIC matching-adjusted indirect comparison, NHT novel hormonal therapy, NIRA + AAP niraparib plus abiraterone acetate, OS overall survival, PSA prostate specific antigen, SMD standardized mean difference, TALA + ENZA talazoparib plus enzalutamide.

Prior to adjustment, the HR for TALA + ENZA versus NIRA + AAP was 0.526 (95% CI: 0.382, 0.726; *p* = 0.0001) for rPFS. In the primary analysis, TALA + ENZA significantly prolonged rPFS (HR: 0.460; 95% CI: 0.280, 0.754; *p* = 0.0021) compared to NIRA + AAP (Fig. 3a and Supplementary Fig. 10). Both sensitivity analyses that incrementally and sequentially adjusted for baseline covariates mutually reported in both trials and exploratory analyses showed consistent results with the primary analysis (Fig. 3a). Additional MAIC results (TALA + ENZA vs AAP + PBO, ENZA vs NIRA + AAP and ENZA vs AAP + PBO) are available in Appendix H.

#### Overall survival

Prior to adjustment, the HR for TALA + ENZA versus NIRA + AAP was 0.580 (95% CI: 0.398, 0.847; *p* = 0.005) for OS. In the primary analysis, the estimated treatment effect was in favor of TALA + ENZA (HR: 0.601; 95% CI: 0.347, 1.041; *p* = 0.069) compared to NIRA + AAP but not statistically significant (Fig. 3b and Supplementary Fig. 11). Both sensitivity analyses that incrementally and sequentially adjusted for baseline covariates mutually reported in both trials and exploratory analyses showed consistent results with the primary analysis (Fig. 3b). Additional MAIC results (TALA + ENZA vs AAP + PBO, ENZA vs NIRA + AAP and ENZA vs AAP + PBO) are available in Appendix H.

#### Objective response rate

Prior to adjustment, the OR for TALA + ENZA versus NIRA + AAP was 1.579 (95% CI: 0.795, 3.135; *p* = 0.192) for ORR. In the primary analysis, the estimated treatment effect was in favor of TALA + ENZA (OR: 1.524; 95% CI: 0.579, 4.016; *p* = 0.394) compared to patients treated with NIRA + AAP but not statistically significant (Supplementary Fig. 6). Both sensitivity analyses that incrementally and sequentially adjusted for baseline covariates mutually reported in both trials and exploratory analyses showed consistent results with the primary analysis (Supplementary Fig. 6).

## Discussion

In the absence of head-to-head RCTs, the present analyses assessed the effectiveness of TALA + ENZA versus 1 L PARPi with NHT in the treatment of mCRPC. The MAIC results suggest TALA + ENZA may provide additional benefit in prolonging rPFS and achieving PSA response while providing similar efficacy across other clinically important endpoints when compared with OLAP + AAP in all-comers. Similarly, when compared to NIRA + AAP in HRRm patients, TALA + ENZA may provide additional benefit in prolonging rPFS and time to PSA progression while numerical improvements were observed for all other outcomes.

TALA + ENZA has demonstrated favorable survival outcomes when compared with key comparator treatments for 1 L mCRPC in the MAIC analysis; however, the limited number of events reported to date resulted in low power for the OS analysis. As of DCO 28/03/23 for TALAPRO-2 Cohort 1, median survival was not reached after a median follow-up duration of 35.8 months in the TALA + ENZA arm. In PROpel (DCO 12/10/22), median survival was 42.1 months after a median follow-up duration of 36.6 months in the OLAP + AAP arm [13]. For TALAPRO-2 Cohort 2 (HRRm patients; DCO 03/10/22), median survival was not reached after a median follow-up duration of 22.2 months in the TALA + ENZA arm, while in MAGNITUDE (DCO 17/06/2022) median survival was 29.3 months after a median follow-up duration of 26.8 months in the NIRA + AAP arm [10].

A key strength of the current analysis was the incorporation of IPD from the TALAPRO-2 trial, which was adjusted according to key comparator populations to facilitate treatment comparisons involving TALA + ENZA and the comprehensive evaluation of cross-trial heterogeneity and potential sources of bias conducted a priori. For instance, consistency of outcome definitions (i.e., defined by RECIST 1.1 or PCWG3) and their method of assessment (BICR versus investigator) was ensured prior to analyses to facilitate fair comparisons and to minimize bias in the results. Furthermore, although MAICs rely on published summary-level data from the comparator trial, the simulation of IPD from published Kaplan–Meier curves from PROpel and MAGNITUDE using the validated Guyot method enabled the reliable reconstruction of the observed IPD. The Kaplan-Meier curves based on simulated IPD were nearly identical to the published Kaplan–Meier curves, with potential slight discrepancies in terms of the exact timings of censoring expected to have only a minimal impact on the estimated HRs.

This analysis is not without limitations. In general, some heterogeneity was observed across the identified trials; however, the comparator trials were considered sufficiently similar to TALAPRO-2, thereby suggesting MAICs between TALAPRO-2 and each of PROpel and MAGNITUDE were feasible, with the important caveat that it was not possible to account for *all* differences between the trial populations using this approach. For instance, as all three trials are currently ongoing, variations in follow-up duration among the study populations may affect the accuracy of outcome assessments and introduce bias. As indicated above, the follow-up times between study population for the DCO dates used in these analyses were quite similar. But we acknowledge that there remain some differences despite our best efforts to control it. We were limited to the evidence that was publicly available to us at the time of our analyses. Furthermore, differences in duration of follow-up do not bias the HR estimates, as the time-to-event endpoints (rPFS, OS, PFS2, time to PSA progression, time to cytotoxic chemotherapy initiation) were analyzed using Cox proportional hazards models. Patients still at risk at the time of data cut were censored, where such administrative censoring is non-informative and does not influence the HR estimates. On the other hand, despite our best efforts to use similar trial follow-up time, these differences may have an impact on objective response rate but are not expected to have a major impact on the conclusions. An update of these analyses should be considered once mature data becomes available. Furthermore, it is plausible that therapies post-discontinuation of study treatment may influence the OS results, but at this time it is unclear the direction and magnitude of this influence. Analyses between TALAPRO-2 and comparator trials were also hindered by a limited reporting of baseline patient characteristics of interest in the comparator trials and a lack of a common comparator lending to unanchored analyses. Additionally, the ability to account for inter-trial differences was further limited by those characteristics for which eligibility criteria were broader in the comparator trials than in TALAPRO-2. Fortunately, for the majority of the key ranked covariates identified, multivariable adjustments in the analysis were possible. Although these MAICs adjusted for observed baseline differences between trials, they are comparisons of non-randomized treatment groups and may therefore be biased by potential unobserved or residual confounding (i.e., differences in patient characteristics, disease severity, concomitant treatments, etc.). The potential influence of molecular subgroups, such as BRCA-positive patients, on treatment response cannot be overlooked [27]. Fazekas et al., meta-analysis suggests that *BRCA*-positive patients are more sensitive to the treatment effect of enzalutamide over abiraterone acetate, resulting in more favorable outcomes for those treated with enzalutamide [27]. Two recent retrospective cohort studies have also suggested better overall survival with enzalutamide-treated patients compared with abiraterone acetate-treated in first-line mCRPC in an overall population that was not molecularly selected [28, 29]. Indeed, in our analyses, as shown in Table 2, the PROpel trial has a higher proportion of patients with *BRCA1* and *BRCA2* mutations compared to TALAPRO-2 before any adjustments (*BRCA1*: 2.3% vs. 0.7% and *BRCA2*: 9.5% vs. 5.0%, PROpel vs TALAPRO-2, respectively). Similarly, as shown in Table 4, the MAGNITUDE trial has a higher proportion of patients with *BRCA1* and *BRCA2* mutations compared to TALAPRO-2 before any adjustments (*BRCA1*: 5.7% vs. 4.5% and *BRCA2*: 40.6% vs. 31.2%, MAGNITUDE vs TALAPRO-2, respectively). After adjusting for the proportion of patients with *BRCA1* and *BRCA2* mutations in the exploratory analysis, such that the proportions are the same for each pairwise comparison (i.e., TALAPRO-2 versus PROpel [Table 2] and TALAPRO-2 versus MAGNITUDE [Table 4]), the results in general, marginally improve in favor of TALA + ENZA combination therapy across several endpoints, suggesting that there may be a complex interaction between specific molecular markers/phenotypes and androgen receptor signalling inhibitors (ARSIs). This is an area of keen interest, and future prospective interventional studies comparing these agents i.e., ARSIs in molecular subgroups are needed for providing higher-level of evidence and to gain a deeper understanding of their impact on therapeutic outcomes. Only a subset of patients in the comparator trials were assessed for ORR based on the reported outcome definitions i.e., only patients with measurable disease at baseline were assessed for ORR. Since baseline characteristics specific to this group of patients was not available in PROpel or MAGNITUDE, patient weight from the entire trial population was used in the ORR analysis for both trials. Furthermore, it is important to recognize that there are challenges in using intermediate clinical trial endpoints, and controversies exist in the literature regarding their validation as surrogates. Additional limitations include a reduced ESS once matching and adjusting have been performed, and the need to transpose to different IPD populations for each analysis. Ultimately, the use of IPD and MAIC can improve the reliability of the indirect comparisons compared with using aggregate data only.

Comparisons of safety outcomes were considered in these analyses; however, there were several challenges in adjusting for the differences in baseline characteristics. Reported prognostic factors and treatment-effect modifiers are not usually predictive of safety outcomes. Additionally, based on clinical opinion from a clinician experienced in treating mCRPC, there are no clear risk factors associated with toxicity from PARPi. As a result, adjusting for differences in baseline characteristics was not feasible and MAICs could not be used to assess safety outcomes. Nonetheless, it is important to consider that efficacy is one of a multitude of factors which play a role in a patient’s choice of therapy. Therapy efficacy, safety, and tolerability to minimize risks and side effects; convenience and preferred method of administration; readiness and urgency to begin treatment; access to treatment based on geographic distribution, national regulations, cost, and insurance restrictions; and likelihood of adherence are all factors considered when choosing the appropriate treatment.

## Conclusion

TALA + ENZA demonstrated favorable results for several clinical outcomes studied, and these were robust across sensitivity analyses. However, potential biases and inherent limitations associated with the complexity of the analyses must be considered. While TALA + ENZA shows promise as a 1 L treatment option for patients with mCRPC, further analysis with mature data is necessary to confirm these findings.

## Supplementary information

Supplementary Material

## References

[1] Sung H, Ferlay J, Siegel RL, Laversanne M, Soerjomataram I, Jemal A, et al. Global cancer statistics 2020: GLOBOCAN estimates of incidence and mortality worldwide for 36 cancers in 185 countries. CA: a cancer J Clinicians. 2021;71:209–49. 10.3322/caac.21660 33538338

[2] Sumanasuriya S, De Bono J. Treatment of advanced prostate cancer—A review of current therapies and future promise. Cold Spring Harb Perspect Med. 2018;8:a030635. 29101113 10.1101/cshperspect.a030635 PMC5983161

[3] Talazoparib + Enzalutamide vs. Enzalutamide Monotherapy in mCRPC (TALAPRO-2) [updated December 6, 2021. Available from: https://clinicaltrials.gov/ct2/show/NCT03395197.

[4] Agarwal N, Azad AA, Carles J, Fay AP, Matsubara N, Heinrich D, et al. Talazoparib plus enzalutamide in men with first-line metastatic castration-resistant prostate cancer (TALAPRO-2): a randomised, placebo-controlled, phase 3 trial. Lancet. 2023;402:291–303. 37285865 10.1016/S0140-6736(23)01055-3

[5] Agarwal N, Azad A, Carles J, Fay AP, Matsubara N, Heinrich D, et al. TALAPRO-2: Phase 3 study of talazoparib (TALA)+ enzalutamide (ENZA) versus placebo (PBO)+ ENZA as first-line (1L) treatment in patients (pts) with metastatic castration-resistant prostate cancer (mCRPC). Am. Society Clinical Oncol.; 2023;41:5004.

[6] Shamseer L, Moher D, Clarke M, Ghersi D, Liberati A, Petticrew M, et al. Preferred reporting items for systematic review and meta-analysis protocols (PRISMA-P) 2015: elaboration and explanation. Bmj. 2015;349:g7647. 10.1136/bmj.g7647 25555855

[7] DistillerSR. Version 2.35. DistillerSR Inc. https://www.distillersr.com/. Accessed September 2021-December 2022.

[8] Clarke NW, Armstrong AJ, Thiery-Vuillemin A, Oya M, Shore N, Loredo E, et al. Abiraterone and olaparib for metastatic castration-resistant prostate cancer. NEJM Evid. 2022;1:EVIDoa2200043. 38319800 10.1056/EVIDoa2200043

[9] Saad F, Clarke NW, Oya M, Shore N, Procopio G, Guedes JD, et al. Olaparib plus abiraterone versus placebo plus abiraterone in metastatic castration-resistant prostate cancer (PROpel): final prespecified overall survival results of a randomised, double-blind, phase 3 trial. Lancet Oncol. 2023;24:1094–1108. 10.1016/S1470-2045(23)00382-0 37714168

[10] Chi K, Sandhu S, Smith M, Attard G, Saad M, Olmos D, et al. Niraparib plus abiraterone acetate with prednisone in patients with metastatic castration-resistant prostate cancer and homologous recombination repair gene alterations: second interim analysis of the randomized phase III MAGNITUDE trial. Annals Oncol. 2023;34:772–82. 10.1016/j.annonc.2023.06.009 PMC10849465 37399894

[11] Moher D, Shamseer L, Clarke M, Ghersi D, Liberati A, Petticrew M, et al. Preferred reporting items for systematic review and meta-analysis protocols (PRISMA-P) 2015 statement. Syst Rev. 2015;4:1–9. 25554246 10.1186/2046-4053-4-1 PMC4320440

[12] NICE. Quality assessment of the relevant clinical effectiveness evidence [updated April 1, 2017. Available from: https://www.nice.org.uk/process/pmg24/chapter/clinical-effectiveness#quality-assessment-of-the-relevant-clinical-effectiveness-evidence.

[13] Clarke NW, Armstrong AJ, Thiery-Vuillemin A, Oya M, Shore ND, Procopio G, et al. Final overall survival (OS) in PROpel: Abiraterone (abi) and olaparib (ola) versus abiraterone and placebo (pbo) as first-line (1L) therapy for metastatic castration-resistant prostate cancer (mCRPC). Am Society Clinical Oncol. 2023;41:LBA16.

[14] Saad F, Armstrong A, Thiery-Vuillemin A, Oya M, Shore N, Procopio G, et al. 1357O Biomarker analysis and updated results from the Phase III PROpel trial of abiraterone (abi) and olaparib (ola) vs abi and placebo (pbo) as first-line (1L) therapy for patients (pts) with metastatic castration-resistant prostate cancer (mCRPC). Ann Oncol. 2022;33:S1160.

[15] Chi KN, Rathkopf D, Smith MR, Efstathiou E, Attard G, Olmos D, et al. Niraparib and abiraterone acetate for metastatic castration-resistant prostate cancer. J Clin Oncol. 2023;41:3339–51. 36952634 10.1200/JCO.22.01649 PMC10431499

[16] Efstathiou E, Smith MR, Sandhu S, Attard G, Saad M, Olmos D, et al. Niraparib (NIRA) with abiraterone acetate and prednisone (AAP) in patients (pts) with metastatic castration-resistant prostate cancer (mCRPC) and homologous recombination repair (HRR) gene alterations: Second interim analysis (IA2) of MAGNITUDE. Am. Society Clinical Oncol. 2023;41:170. 10.1016/j.annonc.2023.06.009 PMC10849465 37399894

[17] Guyot P, Ades A, Ouwens MJ, Welton NJ. Enhanced secondary analysis of survival data: reconstructing the data from published Kaplan-Meier survival curves. BMC Med Res Methodol. 2012;12:1–13. 22297116 10.1186/1471-2288-12-9 PMC3313891

[18] Phillippo D, Ades T, Dias S, Palmer S, Abrams KR & Welton N. NICE DSU Technical Support Document 18: Methods for population-adjusted indirect comparisons in submissions to NICE. NICE Decision Support Unit, ScHARR, University of Sheffield. 2016; Retrieved from https://research-information.bris.ac.uk/ws/portalfiles/portal/94868463/Population_adjustment_TSD_FINAL.pdf.

[19] Saluja R, Cheng S, delos Santos KA, Chan KK. Estimating hazard ratios from published Kaplan‐Meier survival curves: a methods validation study. Res Synth methods. 2019;10:465–75. 31134735 10.1002/jrsm.1362

[20] Armstrong AJ, Lin P, Higano CS, Sternberg CN, Sonpavde G, Tombal B, et al. Development and validation of a prognostic model for overall survival in chemotherapy-naïve men with metastatic castration-resistant prostate cancer. Ann Oncol. 2018;29:2200–7. 30202945 10.1093/annonc/mdy406 PMC6888025

[21] Austin PC. Balance diagnostics for comparing the distribution of baseline covariates between treatment groups in propensity‐score matched samples. Stat Med. 2009;28:3083–107. 19757444 10.1002/sim.3697 PMC3472075

[22] Phillippo DM, Ades AE, Dias S, Palmer S, Abrams KR, Welton NJ. Methods for population-adjusted indirect comparisons in health technology appraisal. Med Decis Mak. 2018;38:200–11. 10.1177/0272989X17725740 PMC5774635 28823204

[23] Signorovitch JE, Wu EQ, Yu AP, Gerrits CM, Kantor E, Bao Y, et al. Comparative effectiveness without head-to-head trials: a method for matching-adjusted indirect comparisons applied to psoriasis treatment with adalimumab or etanercept. Pharmacoeconomics. 2010;28:935–45. 20831302 10.2165/11538370-000000000-00000

[24] Grambsch PM, Therneau TM. Proportional hazards tests and diagnostics based on weighted residuals. Biometrika. 1994;81:515–26.

[25] Signorovitch JE, Sikirica V, Erder MH, Xie J, Lu M, Hodgkins PS, et al. Matching-adjusted indirect comparisons: a new tool for timely comparative effectiveness research. Value Health. 2012;15:940–7. 22999145 10.1016/j.jval.2012.05.004

[26] Phillippo D, Ades T, Dias S, Palmer S, Abrams KR, Welton N NICE DSU technical support document 18: methods for population-adjusted indirect comparisons in submissions to NICE. 2016.

[27] Fazekas T, Szeles AD, Teutsch B, Csizmarik A, Vekony B, Varadi A, et al. Therapeutic sensitivity to standard treatments in BRCA positive metastatic castration-resistant prostate cancer patients—a systematic review and meta-analysis. Prostate Cancer Prostatic Dis. 2023;26:665–72. 36509931 10.1038/s41391-022-00626-2 PMC10638083

[28] George DJ, Ramaswamy K, Yang H, Liu Q, Zhang A, Greatsinger A, et al. Real-world overall survival with abiraterone acetate versus enzalutamide in chemotherapy-naïve patients with metastatic castration-resistant prostate cancer. Prostate Cancer and Prostatic Diseases. 2024:1–9. 10.1038/s41391-024-00816-0 PMC11543594 38538879

[29] Schoen MW, Carson KR, Eisen SA, Bennett CL, Luo S, Reimers MA, et al. Survival of veterans treated with enzalutamide and abiraterone for metastatic castrate resistant prostate cancer based on comorbid diseases. Prostate Cancer Prostatic Dis. 2023;26:743–50. 36104504 10.1038/s41391-022-00588-5 PMC10638085

[30] McGrath S, Zhao X, Steele R, Thombs BD, Benedetti A. Collaboration DSD. Estimating the sample mean and standard deviation from commonly reported quantiles in meta-analysis. Stat methods Med Res. 2020;29:2520–37. 32292115 10.1177/0962280219889080 PMC7390706

[31] Rugo HS, Haltner A, Zhan L, Tran A, Bananis E, Hooper B, et al. Matching-adjusted indirect comparison of palbociclib versus ribociclib and abemaciclib in hormone receptor-positive/HER2-negative advanced breast cancer. J Comp Effectiveness Res. 2021;10:457–67. 10.2217/cer-2020-0272 33626934

