# Supplementary material for: Talazoparib plus enzalutamide versus olaparib plus abiraterone acetate and niraparib plus abiraterone acetate for metastatic castration-resistant prostate cancer: a matching-adjusted indirect comparison
Source: Prostate Cancer Prostatic Dis. 2024 Dec 7;28(3):817–27. doi: 10.1038/s41391-024-00924-x (PMC12399417; doi:10.1038/s41391-024-00924-x)
Supplement: Supplementary file 1 — Supplementary Material [file 41391_2024_924_MOESM1_ESM.docx]

**Supplementary Material**

1. PICOS Criteria

Table 1: PICOS Criteria

|  | **Inclusion Criteria** | **Exclusion Criteria** |
| --- | --- | --- |
| ***Population*** | Study populations or subgroups of patients (humans only; men) with:   - Age ≥18 years - Histologically or cytologically confirmed adenocarcinoma of the prostate - Undergone surgical or medical castration - Metastatic disease - Castration-resistant/Hormone-resistant/Hormone-refractory/Androgen-independent prostate cancer - Asymptomatic or mildly symptomatic^a^ - Treatment naïve in the mCRPC setting | Study populations or subgroups:   - Non-human - Age <18 years - No surgical castration or medical castration - Non-metastatic disease - Non-CRPC - Hormone-sensitive disease - Any previous systemic cancer treatment for mCRPC disease state^b^ |
| ***Interventions*** | - Treatments under investigation for mCRPC provided as a single-agent or a combination treatment - PARP inhibitors (talazoparib, rucaparib, olaparib, niraparib) - Chemotherapy agents   - Taxanes (docetaxel, cabazitaxel)   - Mitoxantrone - Immunotherapies (pembrolizumab, sipuleucel-T) - Novel hormone therapies   - - Direct androgen receptor inhibitors (enzalutamide, apalutamide, darolutamide)     - Abiraterone acetate - Radiotherapy (radium-223) - AKT inhibitor (ipatasertib) | Those not listed in *Inclusion Criteria* |
| ***Comparators*** | - Treatments under investigation for mCRPC provided as a single-agent or combination treatment - PARP inhibitors (talazoparib, rucaparib, olaparib, niraparib) - Chemotherapy agents   - Taxanes (docetaxel, cabazitaxel)   - Mitoxantrone - Immunotherapies (pembrolizumab, sipuleucel-T) - NHTs   - - Direct AR inhibitors (enzalutamide, apalutamide, darolutamide)     - Abiraterone acetate - Radiotherapy (radium-223) - AKT inhibitor (ipatasertib) - BSC or placebo or watchful waiting | Those not listed in *Inclusion Criteria* |
| ***Outcomes*** | - Survival endpoints:   - OS   - PFS   - rPFS   - PFS2 - Response endpoints:   - ORR   - DoR   - PSA response   - Proportion of patients with PSA response ≥50% - Other endpoints:   - Time to PSA progression   - Time to initiation of cytotoxic chemotherapy   - Time to initiation of antineoplastic therapy   - Time to first symptomatic skeletal event   - Opioid use for cancer pain - Safety endpoints:   - Incidence of AEs   - Serious AEs   - AEs leading to discontinuation - Patient-reported outcomes:   - HRQoL (eg, EQ-5D, FACT-P, SF-36, EORTC QLQ-C30, EORTC QLQ-PR25, BPI-SF, PGI-S, etc.) | Those not listed in *Inclusion Criteria* |
| ***Study Design*** | - RCTs irrespective of blinding status^c^   - Phase II, II/III, III - Conference abstracts of RCTs | - Non-RCTs (ie, single-arm clinical trials and observational studies) - Phase I, I/II, IV trials - Open-label extension studies - Pre-clinical studies - Pharmacokinetic studies - Retrospective studies - Case study, series, or reports - Expert opinion articles - Letters - Editorials - Narrative (non-systematic) reviews - Pilot studies - Protocols - SLRs/MAs/NMAs of RCTs^d^ - Non-clinical studies   - Utilities outcomes only   - Economic outcomes only |
| ***Language^e^*** | - Articles in English | All non-English articles |
| ***Dates*** | Databases: inception-present  Conference abstracts: 2019-present | Databases: none  Conference abstracts: prior to 2019 |

^a^ The definition of symptomatic disease will not be restricted, and definitions based on pain and/or other symptoms will be included. Differing definitions will be evaluated at the feasibility assessment stage.

^b^ Androgen deprivation therapy is not exclusionary.

^c^ Crossover trials will only be included if the randomization portion of the trial has relevant data reported.

^d^ Relevant SLRs/MAs/NMAs will not be included in the final included studies list; however, their bibliographies will be reviewed for any additional relevant studies.

^e^ Search strategy will not be limited by language; however, non-English articles will be excluded during the screening phase.

Abbreviations: AEs = adverse events; AKT = protein kinase B; AR = androgen receptor; BPI-SF = Brief Pain Inventory (Short Form); BSC = best supportive care; CRPC = castration-resistant prostate cancer; DoR = duration of response; EORTC QLQ-C30 = European Organisation for Research and Treatment of Cancer Quality of Life of Cancer Patients Questionnaire; EORTC QLQ-PR25 = European Organisation for Research and Treatment of Cancer Quality of Life Questionnaire - Prostate Cancer Module; EQ-5D = EuroQuol 5-Dimension; FACT-P = Functional Assessment of Cancer Therapy-Prostate; HRQoL = health-related quality of life; MA = meta-analysis; mCRPC = metastatic castration-resistant prostate cancer; NMA = network meta-analysis; ORR = objective response rate; OS = overall survival; PARP = poly (ADP-ribose) polymerase; PFS = progression-free survival; PFS2 = progression free survival on next line of therapy; PGI-S = Patient Global Impression of Severity; PSA = prostate-specific antigen; RCT = randomized controlled trial; rPFS = radiographic progression-free survival; SF-36 = 36-Item Short-Form Survey; SLR = systematic literature review.

1. Prognostic Factor Ranking and Stratification Factor Adjustment

***Identification and Rank Ordering of Prognostic Factors***

Confounding driven by differences in baseline patient and disease characteristics that are prognostic of the outcomes of interest leads to biased comparative efficacy estimates if left unadjusted. Twelve potentially important prognostic factors, which were identified based on the previously described literature review, a published analysis on prognostic strength in mCRPC,(1) and by clinical expertise, were ranked in order of importance based on their expected impact on outcome prior to conducting the analysis (**Supplementary Table 2**). The availability of identified prognostic factors in the trials is presented in the **Supplementary Table 3**. The primary analysis adjusted for all factors commonly available in both trials.

***Adjusting for Stratification Factors***

Patients in TALAPRO-2 were stratified at randomization by HRR alteration status (biomarker positive [BM+] versus BM-/unknown) and prior novel hormonal therapy (NHT) or taxane-based chemotherapy in the castration-sensitive PC stage (yes/no). Both PROpel and MAGNITUDE reported these two stratification factors. With respect to HRR alteration status, for the comparison with PROpel, the 21.1% of patients in TALAPRO-2 (n = 85)(2) who were HRR BM+ were adjusted to the 27.8% (n = 111 patients)(3) in PROpel who were HRR BM+. No adjustments were necessary for MAGNITUDE since the HRR BM+ cohorts from both TALAPRO-2 (Cohort 2 ) and MAGNITUDE were used in the analyses. PROpel reported prior NHT and prior taxane-based chemotherapy per Interactive Web Response System (IWRS) as separate variables. MAGNITUDE also reported these variables separately but did not specify based on IWRS or electronic data capture (EDC). TALAPRO-2 IPD differed from comparator trials since it combined these two variables. Prior NHT and prior taxane-based chemotherapy per EDC (rather than IWRS) were reported as separate variables in TALAPRO-2 IPD, aligning with reporting in PROpel and MAGNITUDE. To allow for adjustment of prior therapy stratification factors between TALAPRO-2 and competitor trials, the separated variables per EDC (rather than IWRS) in TALAPRO-2 were used in the analyses. The proportion of patients with stratification factors per EDC versus IWRS were very similar; therefore, using EDC stratification values was considered appropriate.

Table 2: Ranked List of Key Prognostic Factors

| **Rank** | **Identified Factor** |
| --- | --- |
| 1 | Time to mCRPC from continuous ADT (ADT alone or in combination) |
| 2 | Presence of liver metastases |
| 3 | Number of bone metastases (<10 vs >10) |
| 4 | ECOG (0-1 vs 2, 3) |
| 5 | BPI-SF |
| 6 | PSA kinetics or PSA levels in absence of kinetics data |
| 7 | Gleason score |
| 8 | Hemoglobin level |
| 9 | Lactate dehydrogenase level |
| 10 | Albumin level |
| 11 | Alkaline phosphatase level |
| 12 | Neutrophil to lymphocyte ratio |
| Exploratory | BRCA1 |
| Exploratory | BRCA2 |
| Exploratory | BRCA co-occurring alterations |
| Exploratory | PALB2 |

Abbreviations: ADT = androgen deprivation therapy; BPI-SF = Brief Pain Index - Short Form; ECOG = Eastern Cooperative Oncology Group; mCRPC = metastatic castration-resistant prostate cancer; PSA = prostate-specific antigen

1. Adjusted Factors

Table 3: Adjustment of Prognostic Factors for PROpel and MAGNITUDE

| **Rank** | **Identified Factor** | **Available for TALAPRO-2** | **PROpel** | | **MAGNITUDE (HRR BM+)** | |
| --- | --- | --- | --- | --- | --- | --- |
|  |  |  | **Available for PROpel** | **Adjusted Variable** | **Available for MAGNITUDE** | **Adjusted Variable** |
| 1 | Time to mCRPC from continuous ADT | No ^a^ | No ^a^ | No | No | No |
| 2 | Presence of liver metastases | Yes | Yes | Yes | Yes | Yes |
| 3 | Number of bone metastases (<10 vs >10) | Yes ^b^ | Yes ^b^ | Yes | Yes ^b^ | Yes |
| 4 | ECOG (0-1 vs 2, 3) | Yes ^c^ | Yes ^c^ | Yes | Yes ^c^ | Yes |
| 5 | BPI-SF | Yes | Yes | No | Yes | Eligibility criteria |
| 6 | PSA kinetics or PSA levels in absence of kinetics data | Yes | Yes | Yes | Yes | Yes |
| 7 | Gleason score | Yes | Yes | Yes | Yes | Yes |
| 8 | Hemoglobin level | Yes | Yes | Yes | Yes | Yes |
| 9 | Lactate dehydrogenase level | Yes | Yes | Yes | Yes | Yes |
| 10 | Albumin level | Yes | Yes | Yes | No | No |
| 11 | Alkaline phosphatase level | Yes | Yes | Yes | Yes | Yes |
| 12 | Neutrophil to lymphocyte ratio | Yes ^d^ | No | No | No | No |
| Exploratory | BRCA1 | Yes | Yes | Yes | Yes | Yes |
| Exploratory | BRCA2 | Yes | Yes | Yes | Yes | Yes |
| Exploratory | BRCA co-occurring alterations ^e^ | Yes | No | No | Yes | Yes |
| Exploratory | PALB2 | Yes | No | No | Yes | Yes |

^a^ Trial reports median time from initial diagnosis to randomization date.

^b^ Trial reports presence/absence of bone metastases.

^c^ All patients have ECOG 0 or 1.

^d^ Calculated from number of neutrophils and lymphocytes.

^e^ For patients with co-occurring HRR gene alterations, patients were excluded if the co-occurring HRR gene alteration included an HRR alteration that was not tested in MAGNITUDE.

Abbreviations: ADT = androgen deprivation therapy; BPI-SF = Brief Pain Index - Short Form; ECOG = Eastern Cooperative Oncology Group; HRR = homologous recombination repair; mCRPC = metastatic castration-resistant prostate cancer; PSA = prostate-specific antigen

1. Study Quality Assessment

Table 4: Assessment of Study Quality

| **Reference** | **Trial; NCT** | **Was randomization carried out appropriately?** | **Was the concealment of treatment allocation adequate?** | **Were the groups similar at the outset of the study in terms of prognostic factors?** | **Were the care providers, participants, and the outcome assessors blind to treatment allocation?** | **Were there any unexpected imbalances in drop-outs between groups?** | **Is there any evidence to suggest that the authors measured more outcomes than they reported?** | **Did the analysis include an ITT analysis? If so, was this appropriate and were appropriate methods used to account for missing data?** |
| --- | --- | --- | --- | --- | --- | --- | --- | --- |
|  |  |  |  |  |  |  |  |  |
| Agarwal-2023(2) | TALAPRO-2; NCT03395197 | Yes | Yes | Yes | Yes | No | No | Yes |
| Clarke-2022(4) | PROpel; NCT03732820 | Yes | Not clear | Yes | Yes | Not clear | No | Yes |
| Chi-2023(5) | MAGNITUDE;  NCT03748641 | Yes | Yes | Yes | Yes | No | No | Yes |

Abbreviations: ITT = intention-to-treat.

1. Feasibility Assessment

***Trial Design Characteristics***

An overview of the trial characteristics is presented in **Supplementary Table 5.** All trials were phase 3, double-blind, multicenter, and randomized. The experimental arms included poly (ADP-ribose) polymerase inhibitors (PARPi) - talazoparib plus enzalutamide in TALAPRO-2,(2) olaparib plus abiraterone acetate with prednisone/prednisolone in PROpel,(4) and niraparib plus abiraterone acetate with prednisone/ prednisolone in MAGNITUDE.(5) Notably, none of the trials shared a common comparator (i.e., evaluated the same control arm) with TALAPRO-2(2) which evaluated placebo with enzalutamide; PROpel(4) and MAGNITUDE(5) evaluated abiraterone acetate with prednisone/prednisolone as the comparator group.

***Trial Eligibility Criteria***

The patient eligibility criteria are summarized in **Supplementary Table 6**. In terms of study enrolment criteria, factors such as age, Eastern Cooperative Oncology Group (ECOG) performance status, confirmed adenocarcinoma of the prostate, castration status, and metastatic disease in bone were generally similar between the trials. With respect to asymptomatic or mildly symptomatic disease based on pain (brief pain inventory – short form [BPI-SF]), PROpel(4) had no eligibility restrictions based on BPI-SF whereas TALAPRO-2(2) required patients to have a score ≤3 on question 3 of the BPI-SF (worst pain in the last 24 hours). As such, 25.8% of patients in the olaparib plus abiraterone acetate with prednisone/prednisolone arm and 22.7% in the placebo plus abiraterone acetate with prednisone/prednisolone were considered symptomatic (defined as those with a BPI-SF score ≥4 and/or opiate use).(4) Additionally, thresholds for life expectancy differed between PROpel and TALAPRO-2. TALAPRO-2(2) accepted patients with a life expectancy of at least 12 months, whereas PROpel(4) accepted patients with a life expectancy of at least six months. With respect to treatment naïve status, TALAPRO-2(2) required patients to be treatment naïve in the metastatic castration-resistant prostate cancer (mCRPC) stage, whereas MAGNITUDE permitted prior abiraterone acetate therapy in the mCRPC if taken for four months or less.(5) Furthermore, MAGNITUDE focused only on data for a cohort of homologous recombinant repair (HRR) biomarker positive (BM+) patients.(5) Hence, for comparisons with MAGNITUDE, only data from Cohort 2, which reflected the HRR BM+ population from the TALAPRO-2 trial was used in this analysis. MAGNITUDE(5) and PROpel(4) did not allow use of abiraterone acetate in the castration sensitive setting whereas TALAPRO-2(2) did not allow use of enzalutamide in the castration sensitive setting. All three trials allowed the use of taxane-based chemotherapies in the castration sensitive setting.

***Baseline Patient Characteristics***

Baseline patient characteristics were similar between TALAPRO-2 and PROpel, and between TALAPRO-2 and MAGNITUDE with regards to age, proportion with ECOG PS 0-1, proportion with Gleason score ≥8, baseline prostate-specific antigen (PSA) levels, time since initial diagnosis of prostate cancer, and proportion with bone metastases (**Supplementary Table 7**). The proportion of patients who identify as Caucasian was higher for patients in PROpel and MAGNITUDE compared with patients in TALAPRO-2. PROpel patients also had a smaller proportion of patients with BPI-SF pain score ≤3 compared with TALAPRO-2. The trials also differed with respect to how visceral metastases were reported, making between-trial comparisons challenging. The proportion of patients with radiographic evidence of progression was not reported in TALAPRO-2 or PROpel. In PROpel, 25.8% of patients in the olaparib plus abiraterone acetate with prednisone/prednisolone arm and 22.7% in the placebo plus abiraterone acetate with prednisone/prednisolone were considered symptomatic (defined as those with a BPI-SF score ≥4 and/or opiate use).(4) MAGNITUDE did not report time since initial diagnosis of prostate cancer or proportion with radiographic evidence of progression. Lastly, 23.6% in the niraparib plus abiraterone acetate with prednisone/prednisolone group and 22.7% in the placebo plus abiraterone acetate with prednisone/prednisolone group received ≤4 months of abiraterone acetate therapy in the mCRPC stage.(5) Furthermore, MAGNITUDE reported data only for a cohort of HRR BM+ patients.

***Outcome Assessments***

Where reported, outcome characteristics were aligned between the studies. Prior to conducting the analyses, we investigated if outcomes aligned between trials. In all trials, progression in soft tissue was defined per RECIST 1.1 and progression in bone was defined per PCWG3 guidelines. We ensured that these definitions were consistent across trials and considered whether the outcomes were measured by investigators or by blinded independent central review (BICR). PROpel used investigator-assessed rPFS as their primary endpoint; however, they also provided rPFS as assessed by BICR. Only rPFS measured by BICR was used in the analyses presented in the current manuscript to ensure fair comparisons and alignment between trials. By aligning outcome definitions and considering the method of assessment (BICR vs. investigator), we aimed to minimize bias in our results. The definition of rPFS was as follows in TALAPRO-2, PROpel, and MAGNITUDE:

TALAPRO-2: “The time from randomization to first objective evidence of radiographic progression as assessed in soft tissue per RECIST 1.1 or in bone (upon subsequent confirmation) per PCWG3 guidelines by BICR, or death from any cause (occurring within 168 days of treatment discontinuation), whichever occurs first.”

PROpel: “The time from randomization to radiological progression, assessed by independent central review per RECIST 1.1 (soft tissue) and PCWG3 guidelines (bone), or death from any cause, whichever occurs first.”

MAGNITUDE: “The time from randomization to the date of radiographic progression as per BICR evaluated by RECIST 1.1 for progression of soft tissue lesions measured by CT/MRI and PCWG3 for progression by bone lesions observed by bone scan, or death from ay cause, whichever occurs first.”

PROpel did not report time to cytotoxic chemotherapy initiation and MAGNITUDE did not report progression free survival on next line of therapy (PFS2) or PSA response. Furthermore, details on time to PSA progression and objective response rate (ORR) were lacking for MAGNITUDE. Matching-adjusted indirect comparisons (MAIC) were not feasible between TALAPRO-2 and PROpel for the outcome of time to PSA progression because the Kaplan-Meier curve required to perform the analysis was not provided for this outcome in PROpel.

Table 5: Trial Characteristics

| **Trial; NCT** | **Phase** | **Study Design** | **Blinded** | **Setting** | **Geographical Location** | **Treatment arms** | **N randomized** | **Median Follow-up Time (months)** |
| --- | --- | --- | --- | --- | --- | --- | --- | --- |
| **TALAPRO-2; NCT03395197**  (2) | 3 | - Randomized - Parallel Assignment - Comparative | Yes | Multicenter | - North America - Asia - Europe - Oceania - South Africa - South America | Talazoparib 0.5 mg  Enzalutamide 160mg | Cohort 1: 402 | - rPFS: 24.9 ^a^ - OS: 28 ^b^ |
|  |  |  |  |  |  |  | Cohort 2: 200 | - rPFS: 17.5 ^c^ - OS: 22.2 ^c^ |
|  |  |  |  |  |  | Placebo Enzalutamide 160mg | Cohort 1: 403 | - rPFS: 24.6 ^a^ - OS: 27.1 ^b^ |
|  |  |  |  |  |  |  | Cohort 2: 199 | - rPFS: 16.8 ^c^ - OS: 20.2 ^c^ |
| **PROpel; NCT03732820**  (4) | 3 | - Randomized - Parallel Assignment - Comparative | Yes | Multicenter | - North America - Europe - Asia - Australia - South America | Olaparib 300 mg twice daily  Abiraterone acetate 1000mg QD  Prednisone/Prednisolone 5mg BID | 399^d^ | - rPFS, time to PSA response, and AEs: 19.3 ^e^ - ORR: NR ^f^ - OS and PFS2: 36.6 ^g^ |
|  |  |  |  |  |  | Placebo  Abiraterone acetate 1000mg QD  Prednisone/Prednisolone 5mg BID | 397^d^ | - rPFS, time to PSA response, and AEs: 19.2^e^ - ORR: NR ^f^ - OS and PFS2: 36.5 ^g^ |
| **MAGNITUDE; NCT03748641**  (5) | 3 | - Randomized - Parallel Assignment - Comparative | Yes | Multicenter | - North America - Asia - Europe - Australia - South Africa - South America | Niraparib 200 mg once daily  Abiraterone acetate 1000mg QD  Prednisone 10mg QD | 212^h,i^ | - PSA progression, ORR: 18.6 ^j^ - rPFS, OS, TCC, AEs: 26.8 ^k^ |
|  |  |  |  |  |  | Placebo  Abiraterone acetate 1000mg QD  Prednisone 10mg QD | 211^h,i^ |  |

|  | This characteristic is mutually exclusive or very different between TALAPRO-2 and comparator study. |
| --- | --- |

^a^ Based on the full-text publication reporting results at the data cutoff date of August 16, 2022.(2)

^b^ Based on the clinical study report of Cohort 1 provided by Pfizer reporting results at the data cutoff date of March 28, 2023.

^c^ Based on the clinical study report of Cohort 2 provided by Pfizer reporting results at the data cutoff date of October 3, 2022.

^d^ Patients in the PROpel trial consisted of 25.8% in the Olaparib + AAP arm and 20.2% in the placebo + AAP arm who were symptomatic (defined as those with a BPI-SF score ≥4 and/or opiate use)

^e^ Based on the full-text publication reporting results at the primary analysis (data cutoff date of July 30, 2021).(3)

^f^ Based on the full-text publication reporting results at the second interim analysis (data cutoff date of March 14, 2022).(6)

^g^ Based on the presentation from ASCO GU reporting results at the pre-planned final analysis (data cutoff date of October 12, 2022).(7)

^h^ Patients in the MAGNITUDE trial consisted of 23.6% in the Niraparib + AAP arm and 22.7% in the placebo + AAP arm who received <4 months of AAP therapy for first-line mCRPC.

^i^ Patients randomized reflect those within the HRR BM+ cohort as limited data for the HRR BM- cohort was reported.

^j^ Based on the full-text publication reporting results at the first interim analysis (data cutoff date of October 8, 2021).(8)

^k^ Based on the full-text publication reporting results at the second interim analysis (data cutoff date of June 17, 2022).(5)

Abbreviations: AAP = abiraterone acetate; AE = adverse events; BID = twice a day; mCRPC = metastatic castration-resistant prostate cancer; N = number of patients; ORR = objective response rate; OS = overall survival; PSA = prostate-specific antigen; QD = once daily; rPFS = radiographic progression-free survival; TCC = time to cytotoxic chemotherapy.

Table 6: Patient Eligibility Criteria

| **Trial; NCT** | **Age/Status** | **Disease-Related** |
| --- | --- | --- |
| **TALAPRO-2;**  **NCT03395197**(2) | - ≥18 years | - Histologically or cytologically confirmed adenocarcinoma of the prostate |
|  | - ECOG ≤1 | - Asymptomatic or mildly symptomatic mCRPC |
|  | - Life expectancy ≥12 months | - Surgically or medically castrated, with serum testosterone ≤50 ng/dL (≤1.73 nmol/L) |
|  |  | - Metastatic disease in bone documented on bone scan or in soft tissue |
|  |  | - Treatment naive in the mCRPC state. ADT is not exclusionary |
|  |  | - Progressive disease at entry: minimum of 2 rising PSA values, soft tissue disease progression, or bone disease progression |
| **PROpel;**  **NCT03732820**(4)^,^**^a^** | - ≥18 years (≥19 years in South Korea) | - Histologically or cytologically confirmed prostate adenocarcinoma |
|  | - ECOG 0-1 | - No symptomatic criterion reported |
|  | - Life expectancy ≥6 months | - Ongoing androgen deprivation with gonadotropin-releasing hormone analogue or bilateral orchiectomy, with serum testosterone <50ng/dL (2.0 nmol/L) |
|  |  | - Metastatic status defined as at least 1 documented metastatic lesion |
|  |  | - First-line mCRPC |
|  |  | - Documented evidence of progressive disease |
| **MAGNITUDE;**  **NCT03748641**(5)^,^**^b^** | - ≥18 years | - Historically confirmed prostate cancer |
|  | - ECOG 0-1 | - Score of ≤3 on the BPI-SF question number 3 |
|  | - No life expectancy criterion reported | - Castrate levels of testosterone ≤50 ng/dL on a gonadotropin releasing hormone analog or bilateral orchiectomy |
|  |  | - Metastatic disease documented by positive bone scan or metastatic lesions |
|  |  | - No prior systemic therapy, treatment with a PARP inhibitor, or more than 4 months of abiraterone acetate plus prednisone in the mCRPC setting |
|  |  | - No progressive disease criterion reported |

|  | This characteristic is mutually exclusive or very different between TALAPRO-2 and comparator study. |
| --- | --- |

^a^ Patients in the PROpel trial consisted of 25.8% in the Olaparib + AAP arm and 20.2% in the placebo + AAP arm who were symptomatic (defined as those with a BPI-SF score ≥4 and/or opiate use)(4)

^b^ Patients in the MAGNITUDE trial consisted of 23.6% in the Niraparib + AAP arm and 22.7% in the placebo + AAP arm who received <4 months of AAP therapy for first-line mCRPC.(5)
Abbreviations: ADT = androgen deprivation therapy; BPI-SF = Brief Pain Inventory-Short Form; dL = decaliter; ECOG = Eastern Cooperation Oncology Group; L = liter; mCRPC = metastatic castration-resistant prostate cancer; ng = nanogram; nmol = nanomole; PARP = poly (adenosine diphosphate [ADP]-ribose) polymerase; PSA = prostate-specific antigen.

Table 7: Baseline Characteristics

| **Trial;**  **NCT** | **Weighted Average of Active Treatment and Control Arms** | | | | | | | | | | | | |
| --- | --- | --- | --- | --- | --- | --- | --- | --- | --- | --- | --- | --- | --- |
|  | **Age (mean years)** | **Caucasian (%)** | **ECOG PS 0-1 (%)** | **Gleason score ≥8 (%)** | **Baseline PSA (median ng/mL)** | **Time Since Initial Diagnosis (median years)** | **BPI-SF ≤3 (%)** | **Bone Metastases (%)** | **Proportion with Visceral Metastases** | | | | |
|  |  |  |  |  |  |  |  |  | **Total** | **Lung or Liver** | **Node** | **Liver** | **Lung** |
| **TALAPRO-2; NCT03395197**^a^ | 70.6 | 61.9% | 100% | 70.1% | 17.18 | 2.84 | 99.4% | 83.8% | NR | 15.7% | 39% | 3.5% | 13.2% |
| **PROpel; NCT03732820**(4) | 69.5^b^ | 70% | 99.8%^c^ | 65.7% | 17.36^b^ | 3.05 | 74.6% | 86.5% | NR | NR | 31.7%^d^ | 4.1% | 10.3% |
| **MAGNITUDE; NCT03748641**(5) | 69 | 74%^e^ | 100% | 67.9%^e,f^ | 19.40 | NR | 91% | 83% | 21.3% | NR | NR | 7.4% | 10.6% |

^a^ Based on the clinical study report of Cohort 1 provided by Pfizer reporting results at the data cutoff date of March 28, 2023.

^b^ Median value was reported.

^c^ Data missing in 0.3% of patients in each treatment arm.

^d^ PROpel reported distant and locoregional lymph node metastases. The proportion of participants with distant lymph node metastases and locoregional lymph node metastases was 32% and 21%, respectively.

^e^ Based on the full-text publication reporting results at the first interim analysis (data cutoff date of October 8, 2021).(8)

^f^ Characteristics reported at diagnosis.

Abbreviations: BPI-SF = Brief Pain Inventory-Short Form; ECOG = Eastern Cooperative Oncology Group; ng/mL = nanogram/milliliter; NR = not reported; PS = performance status; PSA = prostate-specific antigen.

1. Efficacy Results
   1. TALAPRO-2 versus PROpel
      1. Progression-free Survival on Next Line of Therapy

Prior to adjustment, the HR for TALA+ENZA versus OLAP+AAP was 1.350 (95% CI: 1.025, 1.776; p = 0.032) for PFS2. In the primary analysis, the estimated treatment effect was in favor of OLAP+AAP (HR: 1.143; 95% CI: 0.850, 1.539; p = 0.376) compared to TALA+ENZA but not statistically significant (**Supplementary Figure 1 and 9**). The sensitivity analyses were consistent with the primary analysis (**Supplementary Figure 1**). The exploratory analyses (i.e., incrementally adjusted for *BRCA1* and *BRCA2*) showed consistent results (**Supplementary Figure 1**).

Figure 1: Summary of PFS2 MAIC Results – TALAPRO-2 (TALA+ENZA) and PROpel (OLAP+AAP) (All-comers)


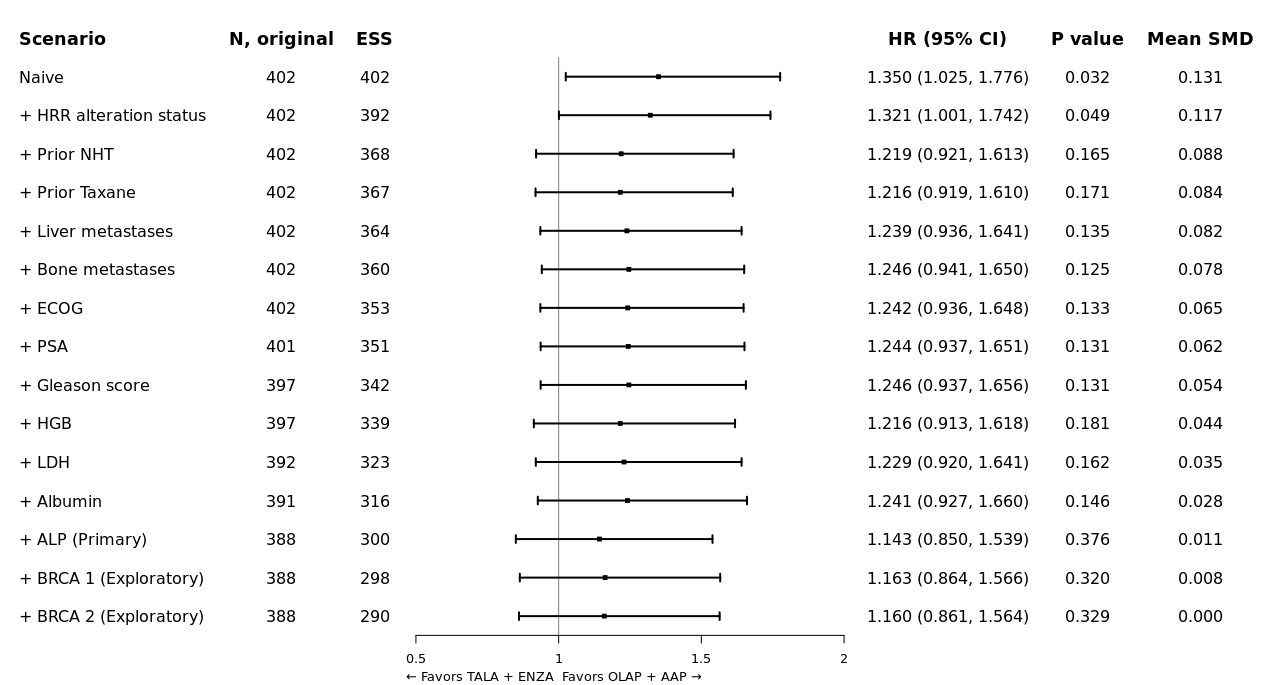


Note: An HR below 1.0 indicates an improved outcome for TALA+ENZA relative to OLAP+AAP.

Note: Results of the naïve analysis and subsequent analyses which adjust for each new characteristic incrementally are shown. The primary analysis is indicated which adjusts for all factors listed above it and ALP. Patients with missing values for a given characteristic were excluded from the corresponding analysis.

Abbreviations: ALP = alkaline phosphatase level; CI = confidence interval; ECOG = Eastern Cooperative Oncology Group; ESS = effective sample size; HGB = hemoglobin level; HR = hazard ratio; HRR = homologous recombination repair; LDH = lactate dehydrogenase level; NHT = novel hormonal therapy; MAIC = matching-adjusted indirect comparison; OLAP+AAP = olaparib plus abiraterone acetate; PFS2 = progression-free survival on next line of therapy; PSA = prostate specific antigen; SMD = standardized mean difference; TALA+ENZA = talazoparib plus enzalutamide.

- - 1. Prostate-specific Antigen Response

Prior to adjustment, the OR for TALA+ENZA versus OLAP+AAP was 1.329 (95% CI: 0.927, 1.907; p = 0.122) for PSA response. In the primary analysis, patients treated with TALA+ENZA were more likely to achieve PSA response (OR: 1.663; 95% CI: 1.101, 2.510; p = 0.016) compared to patients treated with OLAP+AAP (**Supplementary Figure 2**). The sensitivity analyses were consistent with the primary analysis (**Supplementary Figure 2**). The exploratory analyses (i.e., incrementally adjusted for *BRCA1* and *BRCA2*) showed consistent results (**Supplementary Figure 2**).

Figure 2: Summary of PSA Response MAIC Results – TALAPRO-2 (TALA+ENZA) and PROpel (OLAP+AAP) (All-comers)


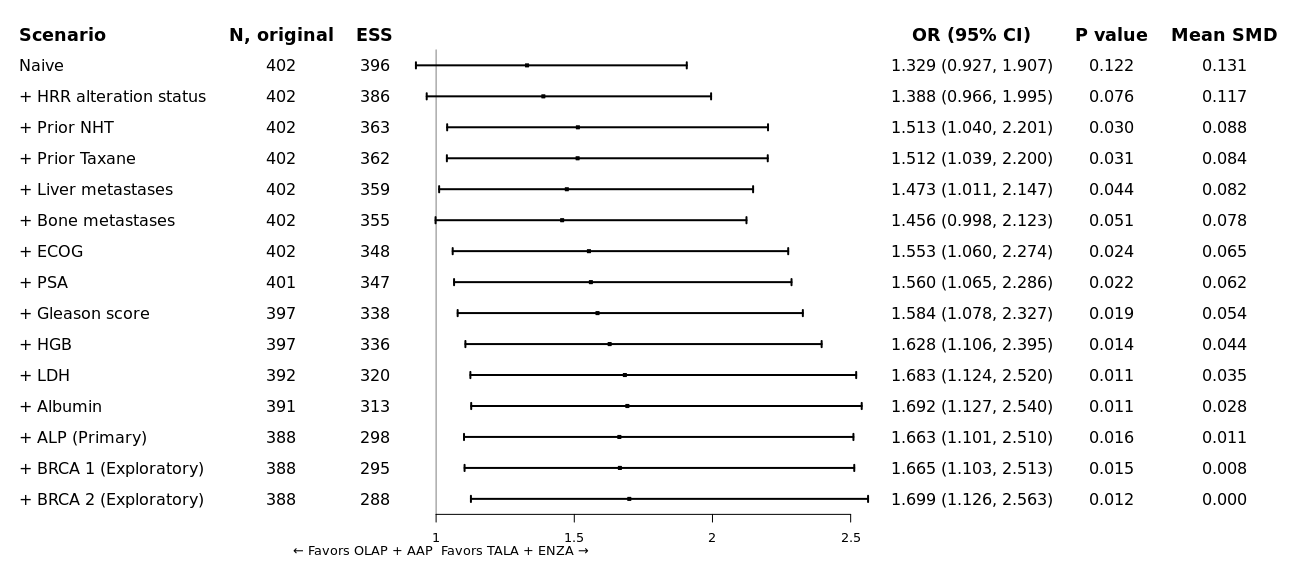


Note: An OR above 1.0 indicates an improved outcome for TALA+ENZA relative to OLAP+AAP.

Note: Results of the naïve analysis and subsequent analyses which adjust for each new characteristic incrementally are shown. The primary analysis is indicated which adjusts for all factors listed above it and ALP. Patients with missing values for a given characteristic were excluded from the corresponding analysis.

Abbreviations: ALP = alkaline phosphatase level; CI = confidence interval; ECOG = Eastern Cooperative Oncology Group; ESS = effective sample size; HGB = hemoglobin level; HRR = homologous recombination repair; LDH = lactate dehydrogenase level; MAIC = matching-adjusted indirect comparison; NHT = novel hormonal therapy; OLAP+AAP = olaparib plus abiraterone acetate; OR = odds ratio; PSA = prostate specific antigen; SMD = standardized mean difference; TALA+ENZA = talazoparib plus enzalutamide.

- - 1. Objective Response Rate

Figure 3: Summary of ORR MAIC Results – TALAPRO-2 (TALA+ENZA) and PROpel (OLAP+AAP) (All-comers)


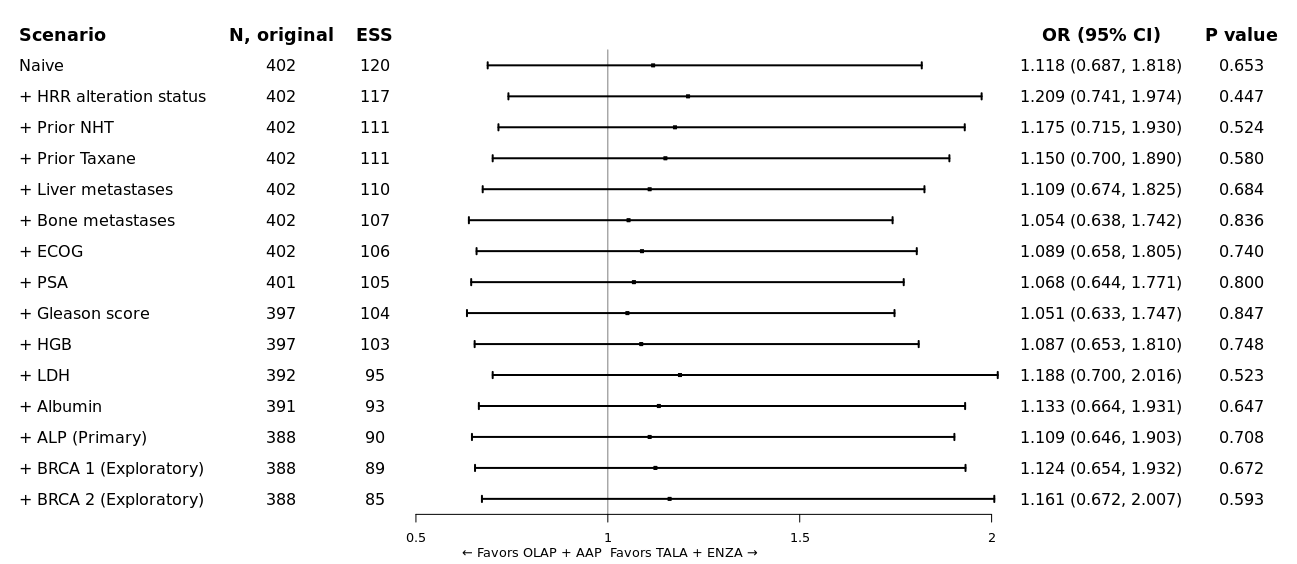


Note: An OR above 1.0 indicates an improved outcome for TALA+ENZA relative to OLAP+AAP.

Note: Results of the naïve analysis and subsequent analyses which adjust for each new characteristic incrementally are shown. The primary analysis is indicated which adjusts for all factors listed above it and ALP. Patients with missing values for a given characteristic were excluded from the corresponding analysis.

Abbreviations: ALP = alkaline phosphatase level; CI = confidence interval; ECOG = Eastern Cooperative Oncology Group; ESS = effective sample size; HGB = hemoglobin level; HRR = homologous recombination repair; LDH = lactate dehydrogenase level; MAIC = matching-adjusted indirect comparison; NHT = novel hormonal therapy; OLAP+AAP = olaparib plus abiraterone acetate; OR = odds ratio; ORR = objective response rate; SMD = standardized mean difference; TALA+ENZA = talazoparib plus enzalutamide.

- 1. TALAPRO-2 versus MAGNITUDE
     1. Time to Cytotoxic Chemotherapy Initiation

Prior to adjustment, the HR for TALA+ENZA versus NIRA+AAP was 0.870 (95% CI: 0.570, 1.328; p = 0.519) for time to cytotoxic chemotherapy initiation. In the primary analysis, the estimated treatment effect was in favor of TALA+ENZA (HR: 0.729; 95% CI: 0.416, 1.278; p = 0.270) compared to NIRA+AAP but not statistically significant (**Supplementary Figure 4 and 12**). The sensitivity analyses were consistent with the primary analysis (**Supplementary Figure 4**). The exploratory analyses (i.e., incrementally adjusted for *BRCA1*, *BRCA2*, *BRCA* co‑occurring, and *PALB2*) showed consistent results (**Supplementary Figure 4**).

Figure 4: Summary of Time to Cytotoxic Chemotherapy Initiation MAIC Results – TALAPRO-2 Cohort 2 (TALA+ENZA) and MAGNITUDE (NIRA+AAP) (HRR BM+)

**
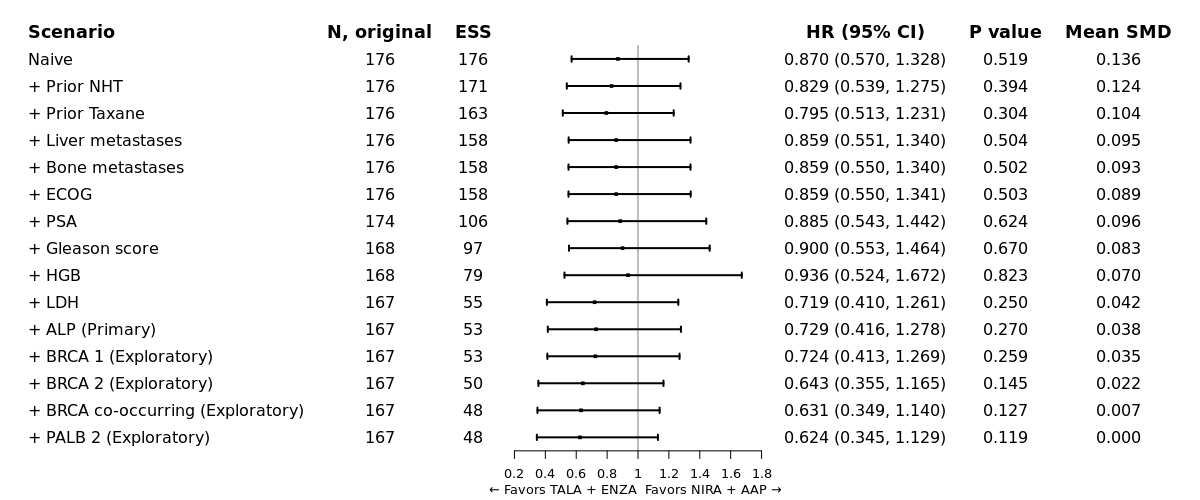
**

Note: An HR below 1.0 indicates an improved outcome for TALA+ENZA relative to NIRA+AAP.
Note: Results of the naïve analysis and subsequent analyses which adjust for each new characteristic incrementally are shown. The primary analysis is indicated which adjusts for all factors listed above it and ALP. Patients with missing values for a given characteristic were excluded from the corresponding analysis.

Abbreviations: ALP = alkaline phosphatase level; CI = confidence interval; ECOG = Eastern Cooperative Oncology Group; ESS = effective sample size; HGB = hemoglobin level; HR = hazard ratio; LDH = lactate dehydrogenase level; MAIC = matching-adjusted indirect comparison; NHT = novel hormonal therapy; NIRA+AAP = niraparib plus abiraterone acetate; PSA = prostate specific antigen; SMD = standardized mean difference; TALA+ENZA = talazoparib plus enzalutamide.

- - 1. Time to PSA Progression

Prior to adjustment, the HR for TALA+ENZA versus NIRA+AAP was 0.598 (95% CI: 0.423, 0.844; p = 0.004) for time to PSA progression. In the primary analysis, patients treated with TALA+ENZA had significantly longer time to PSA progression (HR: 0.578; 95% CI: 0.373, 0.895; p = 0.014) compared to patients treated with NIRA+AAP (**Supplementary Figure 5 and 13**). The sensitivity analyses were consistent with the primary analysis (Supplementary Figure 5). The exploratory analyses (i.e., incrementally adjusted for BRCA1, BRCA2, BRCA co occurring, and PALB2) showed consistent results (**Supplementary Figure 5**).

Figure 5: Summary of Time to PSA Progression MAIC Results – TALAPRO-2 Cohort 2 (TALA+ENZA) and MAGNITUDE (NIRA+AAP) (HRR BM+)


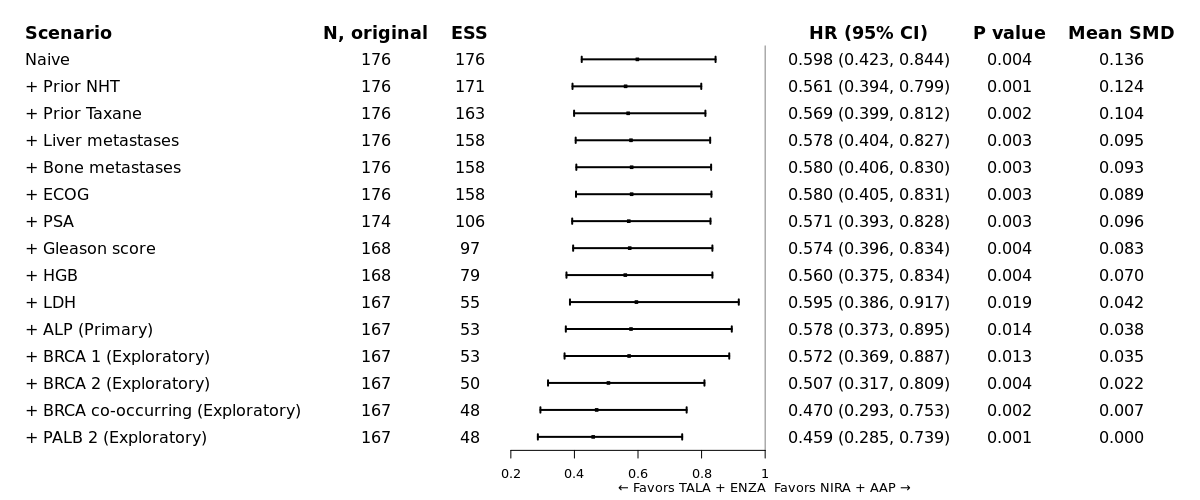


Note: An HR below 1.0 indicates an improved outcome for TALA+ENZA relative to NIRA+AAP.
Note: Results of the naïve analysis and subsequent analyses which adjust for each new characteristic incrementally are shown. The primary analysis is indicated which adjusts for all factors listed above it and ALP. Patients with missing values for a given characteristic were excluded from the corresponding analysis.

Abbreviations: ALP = alkaline phosphatase level; CI = confidence interval; ECOG = Eastern Cooperative Oncology Group; ESS = effective sample size; HGB = hemoglobin level; HR = hazard ratio; LDH = lactate dehydrogenase level; MAIC = matching-adjusted indirect comparison; NHT = novel hormonal therapy; NIRA+AAP = niraparib plus abiraterone acetate; PSA = prostate specific antigen; SMD = standardized mean difference; TALA+ENZA = talazoparib plus enzalutamide.

- - 1. Objective Response Rate

Figure 6: Summary of ORR MAIC Results – TALAPRO-2 Cohort 2 (TALA+ENZA) and MAGNITUDE (NIRA+AAP) (HRR BM+)


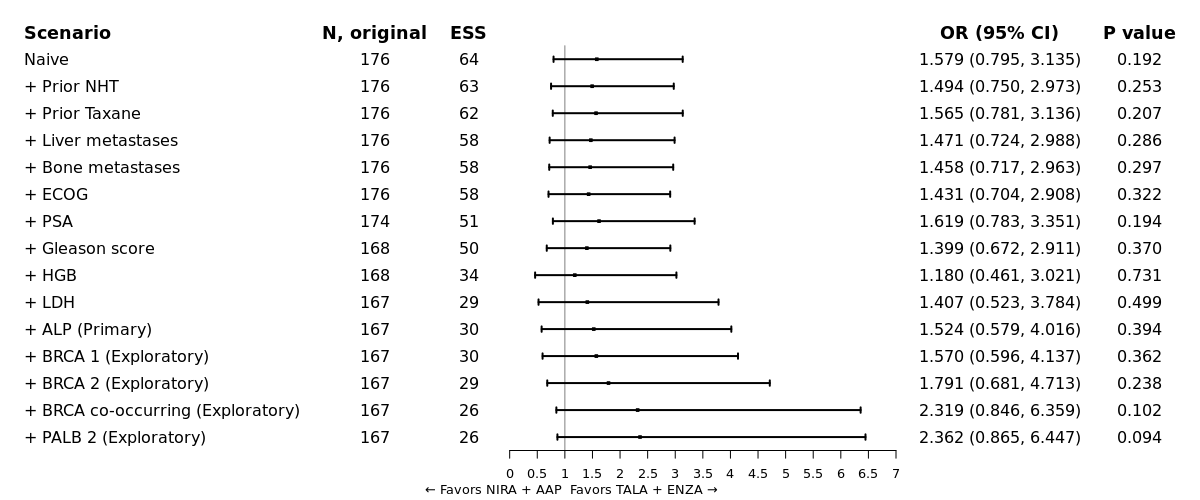


Note: An OR above 1.0 indicates an improved outcome for TALA+ENZA relative to NIRA+AAP.
Note: To align with the definition of ORR in TALAPRO-2 and MAGNITUDE, only patients with measurable disease at baseline were included in the analysis (n=64 for TALAPRO-2 and n=92 for MAGNITUDE).
Note: Results of the naïve analysis and subsequent analyses which adjust for each new characteristic incrementally are shown. The primary analysis is indicated which adjusts for all factors listed above it and ALP. Patients with missing values for a given characteristic were excluded from the corresponding analysis.

Abbreviations: ALP = alkaline phosphatase level; CI = confidence interval; ECOG = Eastern Cooperative Oncology Group; ESS = effective sample size; HGB = hemoglobin level; HR = hazard ratio; LDH = lactate dehydrogenase level; MAIC = matching-adjusted indirect comparison; NHT = novel hormonal therapy; NIRA+AAP = niraparib plus abiraterone acetate; ORR = objective response rate; PSA = prostate specific antigen; SMD = standardized mean difference; TALA+ENZA = talazoparib plus enzalutamide.

1. Kaplan-Meier (KM)-Estimated Efficacy Outcomes

Figure 7: KM-Estimated rPFS (BICR) – TALAPRO-2 (TALA+ENZA) and PROpel (OLAP+AAP) (All-comers)


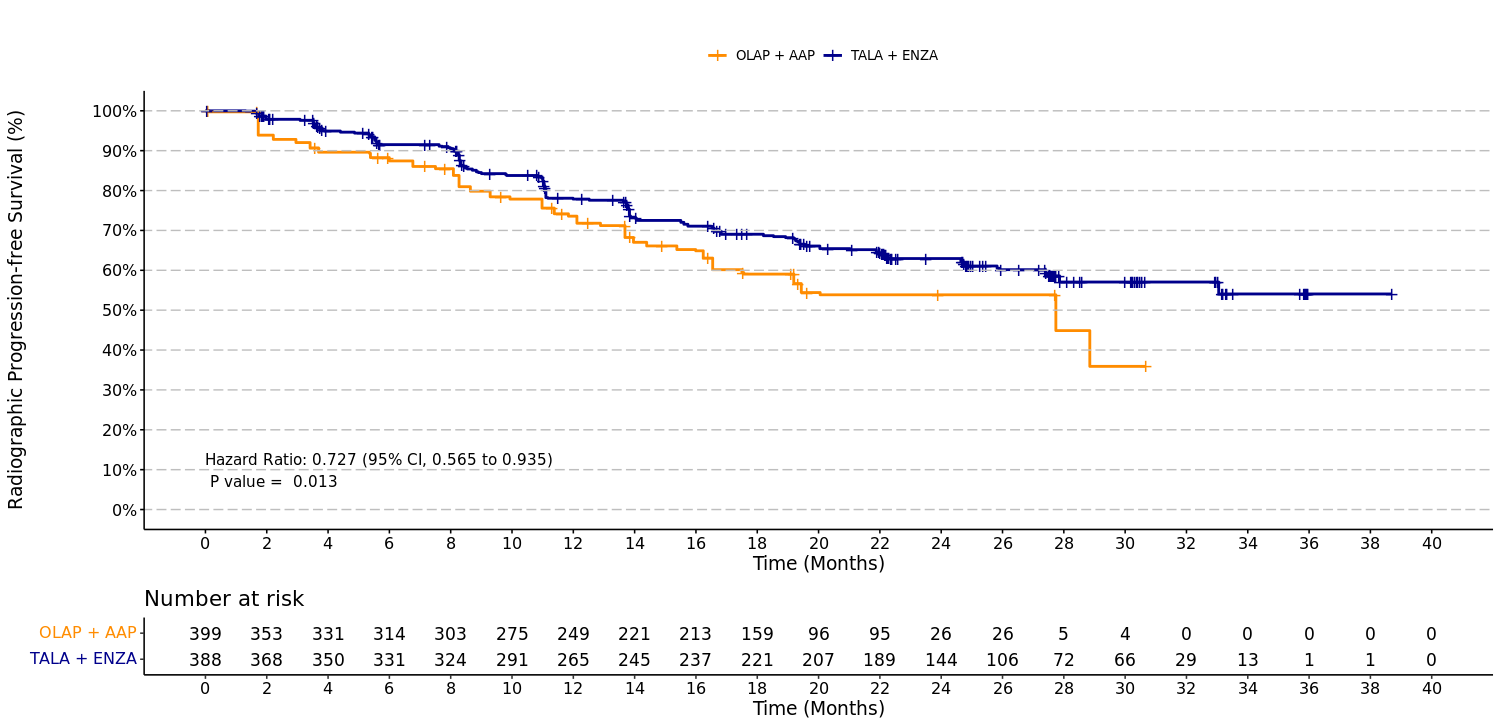
Abbreviations: BICR = blinded independent central review; CI = confidence interval; KM = Kaplan-Meier; OLAP+AAP = olaparib plus abiraterone acetate; rPFS = radiographic progression-free survival; TALA+ENZA = talazoparib plus enzalutamide.

Figure 8: KM-Estimated OS – TALAPRO-2 (TALA+ENZA) and PROpel (OLAP+AAP) (All-comers)


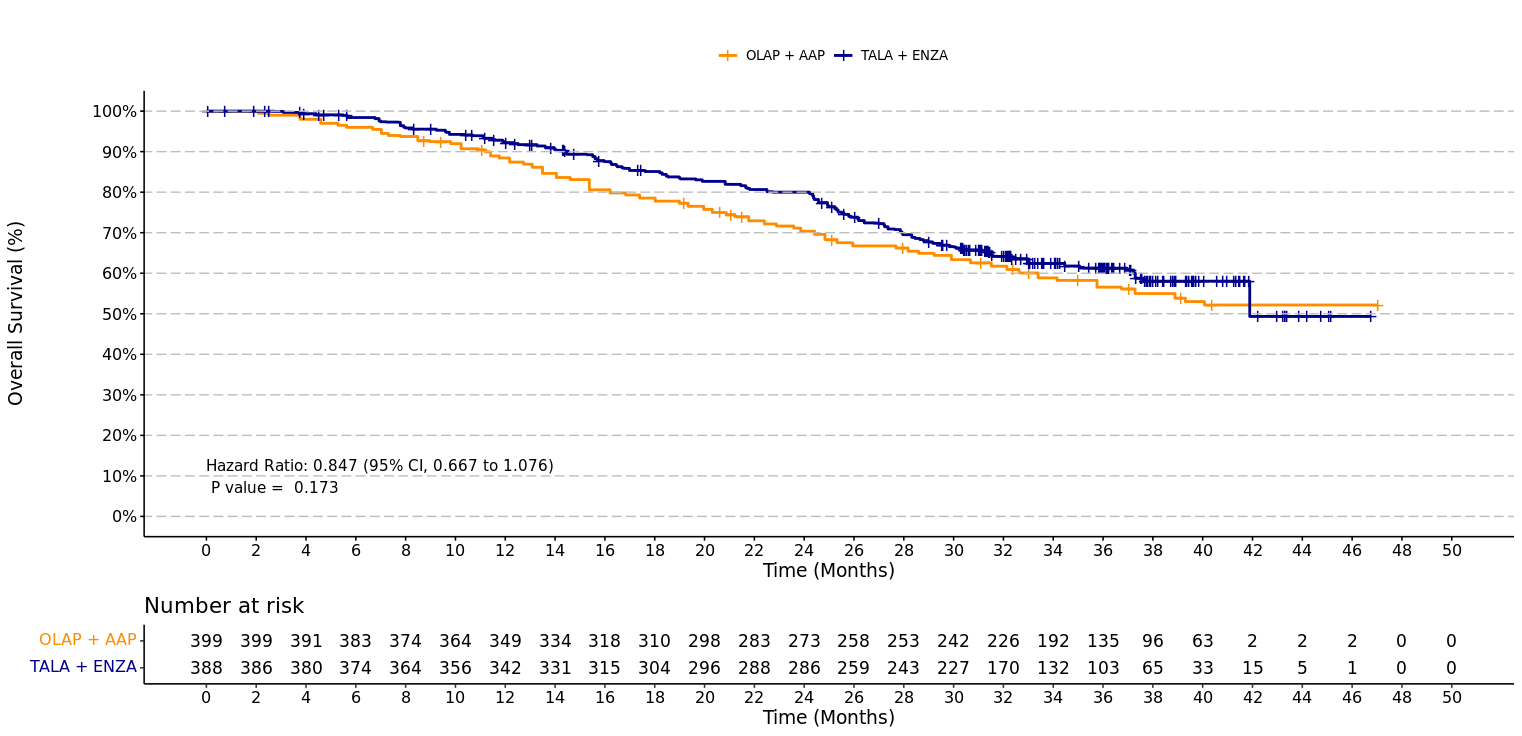
 Abbreviations: CI = confidence interval; KM = Kaplan-Meier; OLAP+AAP = olaparib plus abiraterone acetate; OS = overall survival; TALA+ENZA = talazoparib plus enzalutamide.

Figure 9: KM-Estimated PFS2 – TALAPRO-2 (TALA+ENZA) and PROpel (OLAP+AAP) (All-comers)


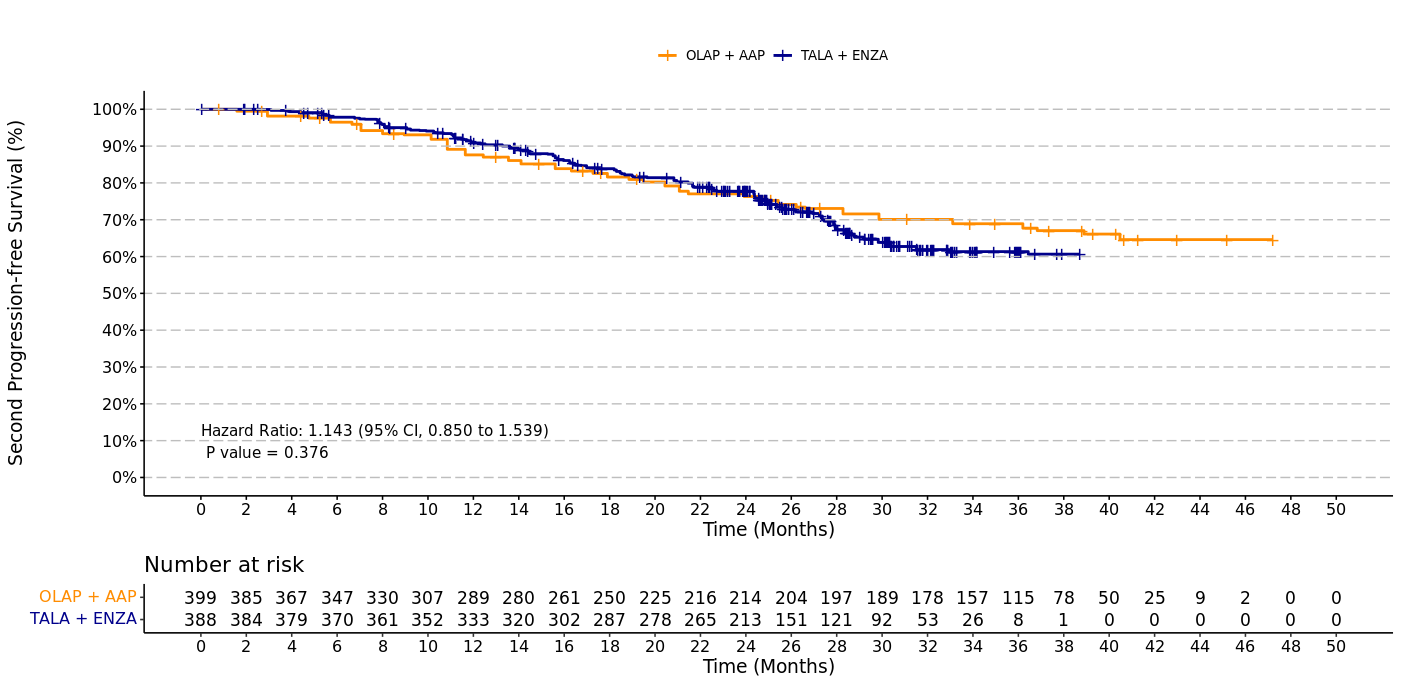
 Abbreviations: CI = confidence interval; KM = Kaplan-Meier; OLAP+AAP = olaparib plus abiraterone acetate; PFS2 = progression-free survival on next line of therapy; TALA+ENZA = talazoparib plus enzalutamide.

Figure 10: KM-Estimated rPFS (BICR) – TALAPRO-2 Cohort 2 (TALA+ENZA) and MAGNITUDE (NIRA+AAP) (HRR BM+)


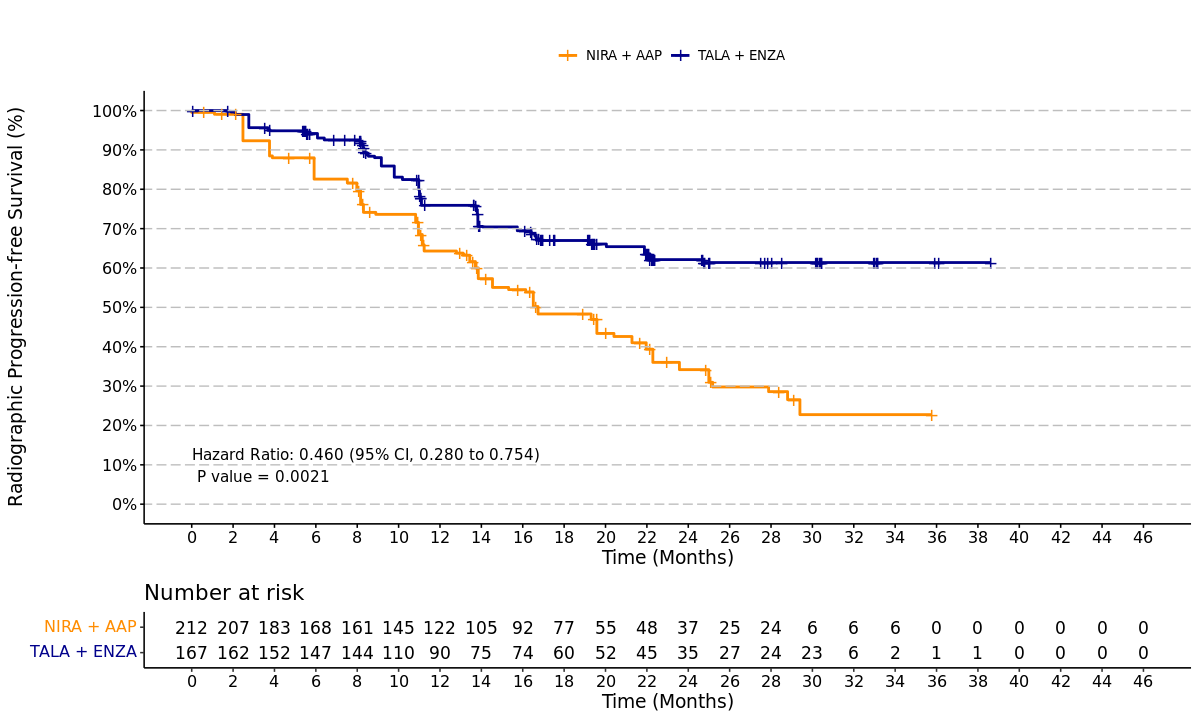
 Abbreviations: BICR = blinded independent central review; CI = confidence interval; KM = Kaplan-Meier; NIRA+AAP = niraparib plus abiraterone acetate; rPFS = radiographic progression-free survival; TALA+ENZA = talazoparib plus enzalutamide.

Figure 11: KM-Estimated OS – TALAPRO-2 Cohort 2 (TALA+ENZA) and MAGNITUDE (NIRA+AAP) (HRR BM+)


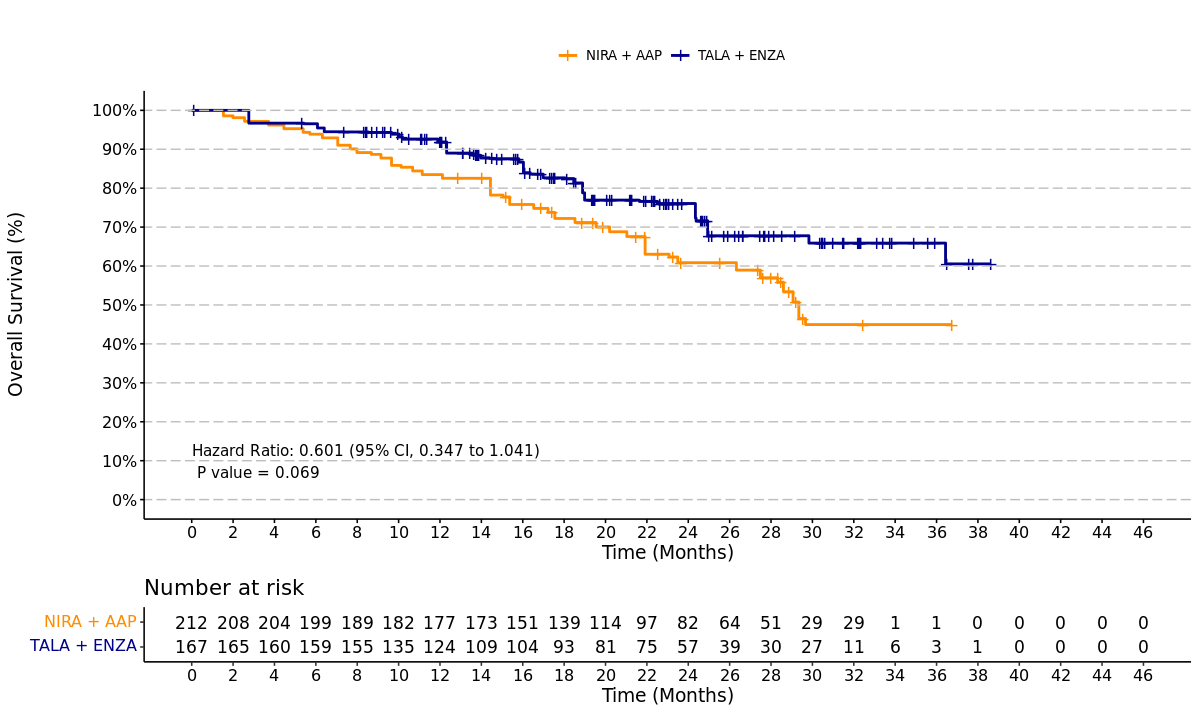
 Abbreviations: CI = confidence interval; KM = Kaplan-Meier; NIRA+AAP = niraparib plus abiraterone acetate; OS = overall survival; TALA+ENZA = talazoparib plus enzalutamide.

Figure 12: KM-Estimated Time to Cytotoxic Chemotherapy Initiation – TALAPRO-2 Cohort 2 (TALA+ENZA) and MAGNITUDE (NIRA+AAP) (HRR BM+)


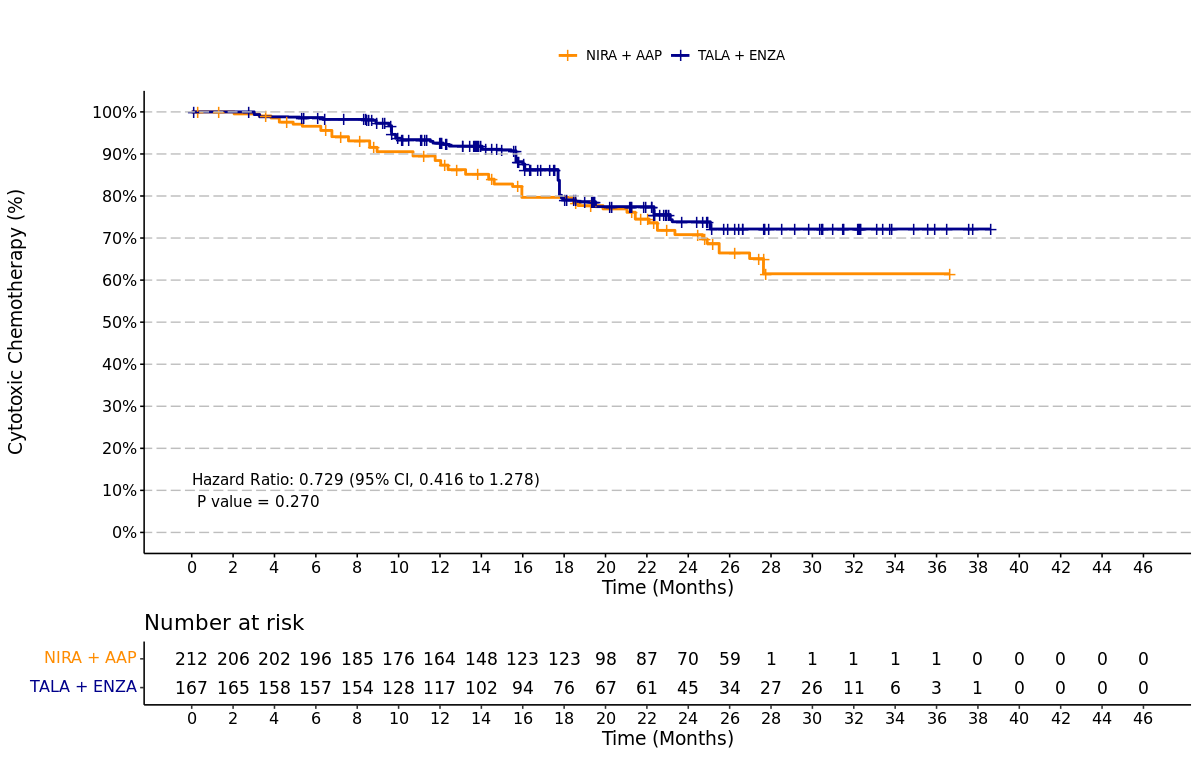
 Abbreviations: CI = confidence interval; KM = Kaplan-Meier; NIRA+AAP = niraparib plus abiraterone acetate; TALA+ENZA = talazoparib plus enzalutamide.

Figure 13: KM-Estimated Time to PSA Progression – TALAPRO-2 Cohort 2 (TALA+ENZA) and MAGNITUDE (NIRA+AAP) (HRR BM+)


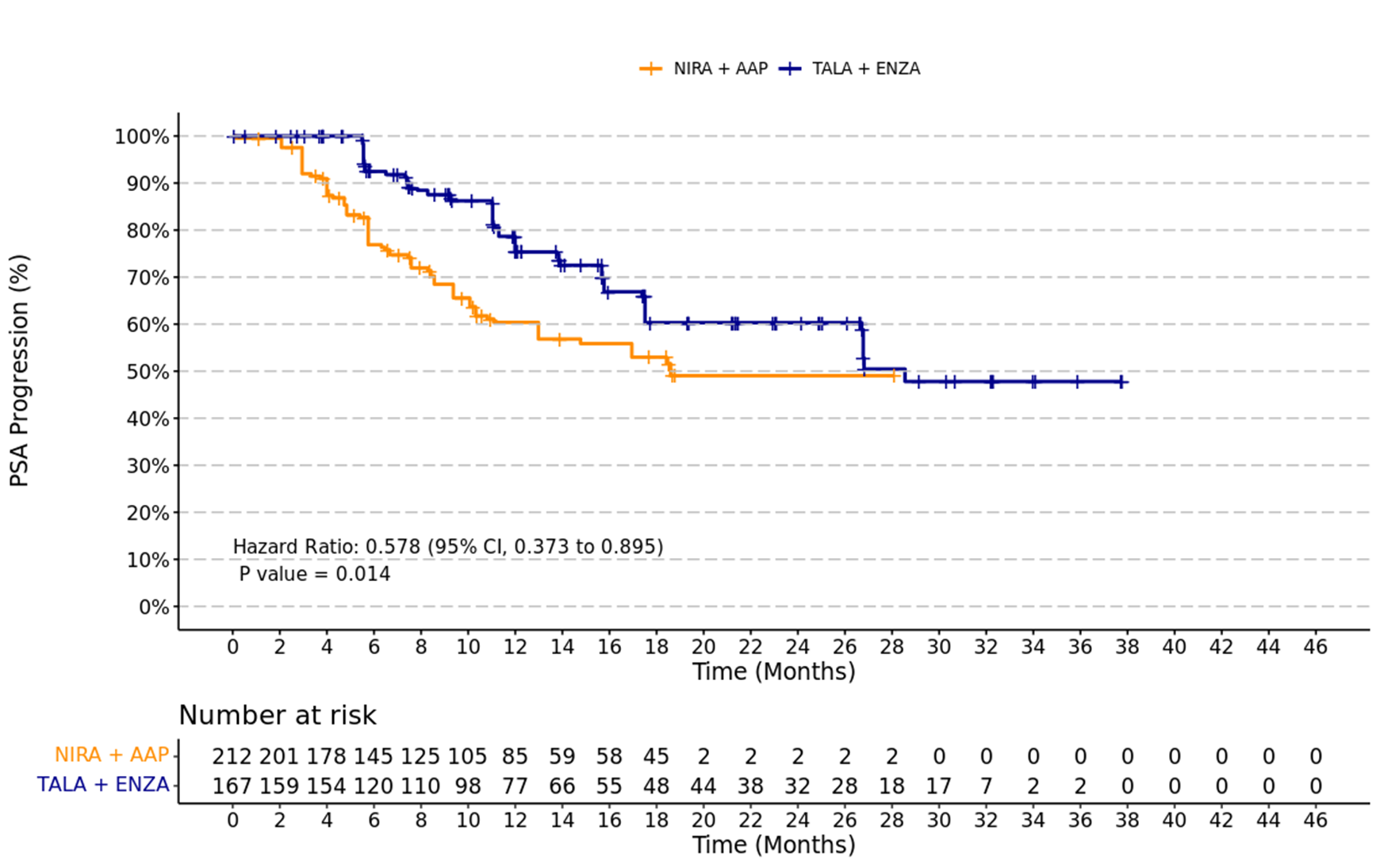
Abbreviations: CI = confidence interval; KM = Kaplan-Meier; NIRA+AAP = niraparib plus abiraterone acetate; PSA = prostate specific antigen; TALA+ENZA = talazoparib plus enzalutamide.

1. Additional MAIC Results

Table 8: Summary of Additional MAIC Results

| **Outcome** | | **ENZA (TALAPRO-2 Cohort 1 All-comers) vs OLAP+AAP (PROpel)** | **ENZA (TALAPRO-2** **Cohort 1 All-comers) vs AAP+PBO (PROpel)** | **TALA+ENZA (TALAPRO-2 Cohort 1 All-comers) vs AAP+PBO (PROpel)** | **ENZA (TALAPRO-2 Cohort 2 HRR BM+) vs NIRA+AAP**  **(MAGNITUDE)** | **ENZA (TALAPRO-2 Cohort 2 HRR BM+) vs AAP+PBO (MAGNITUDE)** | **TALA+ENZA (TALAPRO-2 Cohort 2 HRR BM+) vs AAP+PBO (MAGNITUDE)** |
| --- | --- | --- | --- | --- | --- | --- | --- |
| ***Primary analysis ^a^*** | | | | | | | |
| rPFS (BICR) | HR  (95% CI) | 1.227  (0.976, 1.542) | **0.702**  **(0.565, 0.871)** | **0.421**  **(0.331, 0.536)** | 1.561  (0.921, 2.646) | 0.920  (0.667, 1.269) | **0.397**  **(0.275, 0.573)** |
| OS |  | 1.073  (0.854, 1.349) | 0.826  (0.661, 1.032) | **0.648**  **(0.512, 0.819)** | **0.449**  **(0.210, 0.960)** | **0.546**  **(0.343, 0.870)** | **0.545**  **(0.341, 0.872)** |
| ***Fully adjusted analysis ^b^*** | | | | | | | |
| rPFS (BICR) | HR  (95% CI) | 1.250  (0.993, 1.573) | **0.702**  **(0.565, 0.872)** | **0.413**  **(0.324, 0.527)** | 1.667  (0.942, 2.951) | 0.937  (0.679, 1.293) | **0.323**  **(0.215, 0.485)** |
| OS |  | 1.060  (0.840, 1.336) | 0.809  (0.646, 1.012) | **0.644**  **(0.509, 0.814)** | 0.600  (0.246, 1.462) | **0.496**  **(0.297, 0.829)** | **0.487**  **(0.297, 0.799)** |

Note: An HR below 1.0 indicates an improved outcome for ENZA / TALA+ENZA relative to OLAP+AAP / AAP+PBO / NIRA+AAP / AAP+PBO. Bolded values indicate statistical significance.

^a^ The primary analysis adjusts for all ranked factors available in both trials listed in **Supplementary Table 3**.

^b^ The fully adjusted analysis adjusts for all ranked factors and exploratory factors available in both trials listed in **Supplementary** **Table 3**.

Abbreviations: AAP+PBO = abiraterone + placebo; BICR = blinded independent central review; CI = confidence interval; ENZA = enzalutamide; HR = hazard ratio; HRR BM+ = homologous recombination repair biomarker positive; MAIC = matching-adjusted indirect comparison; NIRA+AAP = niraparib plus abiraterone acetate; OLAP+AAP = olaparib + abiraterone acetate; OS = overall survival; rPFS = radiographic progression-free survival; TALA+ENZA = talazoparib plus enzalutamide.

References

1. Armstrong AJ, Lin P, Higano CS, Sternberg CN, Sonpavde G, Tombal B, et al. Development and validation of a prognostic model for overall survival in chemotherapy-naïve men with metastatic castration-resistant prostate cancer. Annals of Oncology. 2018;29(11):2200-7.

2. Agarwal N, Azad AA, Carles J, Fay AP, Matsubara N, Heinrich D, et al. Talazoparib plus enzalutamide in men with first-line metastatic castration-resistant prostate cancer (TALAPRO-2): a randomised, placebo-controlled, phase 3 trial. The Lancet. 2023.

3. Clarke NW, Armstrong AJ, Thiery-Vuillemin A, Oya M, Shore N, Loredo E, et al. Abiraterone and olaparib for metastatic castration-resistant prostate cancer. NEJM Evidence. 2022;1(9):EVIDoa2200043.

4. Clarke Noel W, Armstrong Andrew J, Thiery-Vuillemin A, Oya M, Shore N, Loredo E, et al. Abiraterone and Olaparib for Metastatic Castration-Resistant Prostate Cancer. NEJM Evidence. 2022;1(9):EVIDoa2200043.

5. Chi K, Sandhu S, Smith M, Attard G, Saad M, Olmos D, et al. Niraparib plus abiraterone acetate with prednisone in patients with metastatic castration-resistant prostate cancer and homologous recombination repair gene alterations: second interim analysis of the randomized phase III MAGNITUDE trial. Annals of Oncology. 2023.

6. Saad F, Armstrong A, Thiery-Vuillemin A, Oya M, Shore N, Procopio G, et al. 1357O Biomarker analysis and updated results from the Phase III PROpel trial of abiraterone (abi) and olaparib (ola) vs abi and placebo (pbo) as first-line (1L) therapy for patients (pts) with metastatic castration-resistant prostate cancer (mCRPC). Annals of Oncology. 2022;33:S1160.

7. Clarke NW, Armstrong AJ, Thiery-Vuillemin A, Oya M, Shore ND, Procopio G, et al. Final overall survival (OS) in PROpel: Abiraterone (abi) and olaparib (ola) versus abiraterone and placebo (pbo) as first-line (1L) therapy for metastatic castration-resistant prostate cancer (mCRPC). American Society of Clinical Oncology; 2023.

8. Chi KN, Rathkopf D, Smith MR, Efstathiou E, Attard G, Olmos D, et al. Niraparib and abiraterone acetate for metastatic castration-resistant prostate cancer. Journal of Clinical Oncology. 2023;41(18):3339-51.
